# Proceedings to the 7^th^ Annual Conference of the Particle Therapy Cooperative Group North America (PTCOG-NA): 18-20 September 2021 Virtual

**DOI:** 10.14338/IJPT-22-PTCOG-NA-8.4

**Published:** 2022-02-18

**Table:** 

Table of Contents	
Program Description	pg. 82
2021 Committees	pg. 82
Oral Presentations	pg. 83
Poster Presentations	pg. 109

## About PTCOG-NA

The Particle Therapy Cooperative Group - North America (PTCOG-NA) is the North American chapter of the international Particle Therapy Cooperative Group. This non-profit professional society has been created in January 2013 to enhance collaboration between its members, create a platform for scientific exchange, and develop treatment guidelines, education, and training initiatives for particle therapy. The society will take steps towards establishing a particle therapy clinical trial collaborative group, as well as instituting representation in industrial technology, particle therapy innovations, involvement in health care politics (as it pertains to particle beam therapy), and improving relationships with other professional societies in radiation oncology. In collaboration with PTCOG, PTCOG-NA helped to establish the *International Journal of Particle Therapy* (*IJPT*).

### Annual Conference Target Audience

Healthcare professionals who treat cancer patients using radiation therapy/particle therapy and specifically:

- Radiation Oncologists
- Medical Physicists
- Dosimetrists
- Residents
- Radiation Therapists

### PTCOG-NA 2021 Committees Executive Committee

**Anita Mahajan, M.D., President**

**Hesham E. Gayar, M.D., Vice-President**

**Bradford Hoppe, M.D., Secretary**

**Carl J. Rossi, M.D., Treasurer**

**Eugen B. Hug, M.D., Past President**

### Conference Planning Committee Members

**Ramesh Rengan, MD, PhD, FASTRO**

*Professor and Chair, Department of Radiation Oncology, University of Washington, and Professor, Clinical Research Division, Fred Hutchinson Cancer Research Center, Seattle, WA*

**Jing Zeng, M.D.**

*Associate Professor, Radiation Oncology, University of Washington, and Medical Director, SCCA Proton Therapy Center, Seattle, WA*

### Oral Abstracts ***Biology: New Applications in Particle Therapy***

## PTCNA-0071

### Effects of particle LET on IFNβ and TREX1 expression

#### Devin Miles^1,2,3^, Yiu-Hsin Chang^3^, Ning Cao^2^, George Sandison^2^, Robert Stewart^2^, Greg Moffitt^2^, Thomas Pulliam^4^, Peter Goff^2^, Paul Nghiem^4^, Keith Stantz^3^

##### ^1^Johns Hopkins University, Radiation Oncology, Baltimore- MD, USA ^2^University of Washington, Radiation Oncology, Seattle- WA, USA ^3^Purdue University, School of Health Sciences, West Lafayette- IN, USA ^4^University of Washington, Dermatology, Seattle- WA, USA

**Purpose**: Cancer cells produce innate immune signals following detection of radiation-induced cytosolic DNA via signaling pathways such as cGAS-STING. High linear energy transfer (LET) radiations induce more DNA double-strand breaks (DSBs) per unit dose than low-LET radiations, potentially enhancing immunogenic effects. This work explores the *in vitro* dose response characteristics of pro-immunogenic interferon-beta (IFNβ) and cGAS-STING antagonist three-prime repair exonuclease 1 (TREX1) from varying-LET radiations.

**Methods**: IFNβ and TREX1 expression were measured in MCC13 cells irradiated with graded doses of x-rays or fast neutrons (comparable LET to carbon-12) via ELISA, immunofluorescence, and qPCR assays. Laboratory measurement of the RBE for IFNβ production (RBE_IFNβ_) and TREX1 upregulation (RBE_TREX1_) was compared to the modeled RBE for DSB induction (RBE_DSB_) from Monte Carlo DNA damage simulations. RBE_IFNβ_ models were applied to radiation transport simulations to quantify the potential secretion of IFNβ from representative proton, helium-4, and carbon-12 beams.

**Results**: Maximum IFNβ secretions occurred at 5.7 Gy and 14.0 Gy for neutrons and x-rays, respectively (RBE_IFNβ_ of 2.5). TREX1 signal increased linearly, with a four-fold higher upregulation per unit dose for fast neutrons (RBE_TREX1_ of 4.0). Monte Carlo modeling suggests an enhanced Bragg peak-to-entrance ratio for IFNb production in charged particle beams.

**Conclusion**: High-LET radiation initiates larger IFNb and TREX1 responses per unit dose than low-LET radiations. RBE_IFNβ_ is comparable to published values for RBE _DSB_, whereas RBE_TREX1_ is roughly twofold higher. Therapeutic advantages of high-LET versus low-LET radiation remain unclear. Potential TREX1-targeted interventions may enable IFNb-mediated immunogenic responses at lower doses of high-LET radiations.

## PTCNA-0073

### Biologic dose escalation in proton lattice radiotherapy plans

#### Hok Seum Wan Chan Tseung^1^, Shima Ito^1^, Yan Zhang^1^, Michael Grams^1^

##### ^1^Mayo Clinic, Radiation Oncology, Rochester MN, USA

**Aim**: To implement lattice radiotherapy using proton pencil beam scanning, and demonstrate treatments that are spatially fractionated in physical dose (PD), with significant escalation of biologic dose (BD) and dose-averaged linear energy transfer (LET_d_) in the vicinity of the high PD regions.

**Method**: For 5 patients with bulky tumors, spatial proton dose fractionation inside the GTV was achieved using proton lattice radiotherapy (pLRT). This involves a 3D lattice of 1.5-cm diameter spherical dose regions separated by 3 cm on average. pLRT plans were created with Eclipse (Varian Medical Systems). Two fields with an opening angle of at least 40 degrees were used to reduce skin dose at entrance. Dose valleys between spheres were kept below 40% of the peak PD. The resulting LET_d_ distributions were calculated with an in-house GPU-based Monte Carlo simulation. BD was estimated from LET_d_ and PD by using published formulae that are based on the linear-quadratic model, as well as a simpler model that assumes a linear relationship between BD and the product of LET_d_ (in keV/μm) and PD: BD=1.1PD(0.08LET_d_+0.88).

**Results**: Within the high dose spheres, peak BD values in excess of 140% of the prescription dose were observed (see figures). LET_d_ values in the spheres reached values greater than 4 keV/μm. This was achieved without using any explicit LET_d_ optimization technique, and is a direct consequence of end-of-range energy deposition within the spheres.

**Conclusion**: Besides spatial fractionation, a feature of pLRT is BD escalation. This can be advantageous for debulking radioresistant or hypoxic tumors.

## PTCNA-0021

### HSP90i by Ganetespib selectively radiosensitizes cancer cells for proximal and distal Spread-Out Bragg peak (SOBP) proton irradiation

#### Simon Deycmar^1,2^, Elisabeth Mara^3,4^, Sylvia Kerschbaum-Gruber^3,5^, Verena Waller^1^, Dietmar Georg^3,5^, Martin Pruschy^1^

##### ^1^University Hospital Zurich, Laboratory of Applied Radiobiology- Dept. of Radiation Oncology, Zurich, Switzerland ^2^Wake Forest School of Medicine, Department of Pathology - Section on Comparative Medicine, Winston-Salem, USA ^3^Medical University of Vienna, Department of Radiation Oncology, Vienna, Austria ^4^University of Applied Science, Na, Wiener Neustadt, Austria ^5^MedAustron Center for Ion Beam Therapy and Research, Non Clinical Research, Wiener Neustadt, Austria

**Background**: This study investigates the radiosensitizing effect of Ganetespib for proton irradiation at a proximal and distal position in a SOBP in comparison to photon irradiation. Rad51, a key protein of homologous recombination repair (HRR), is downregulated by HSP90-inhibiting Ganetespib which provides a promising rational for a specifically proton-sensitizing approach.

**Methods and Materials**: A549 and FaDu cells were treated with low-dose Ganetespib and irradiated with 200kV photons respectively protons at a proximal, low linear energy transfer (LET, 2.1keV/μm) and a distal, higher LET (4.5keV/μm) position within a SOBP. Cellular survival was determined by clonogenic assay, cell cycle distribution by flow cytometry, Rad51 protein levels by western blotting and γH2AX foci by immunofluorescence microscopy.

**Results**: Ganetespib reduced clonogenicity in both cancer cell lines exclusively in response to proton irradiation of both investigated LETs. Upon proton irradiation, a more pronounced accumulation of cells in S/G2/M phase became evident with Ganetespib reducing this population. Rad51 protein levels were more extensively and more persistently elevated in proton- than in photon-irradiated cells and suppressed by Ganetespib at each investigated time point. Immunofluorescence staining demonstrated a similar induction and removal of γH2AX foci independent of Ganetespib which suggests compensation by more error-prone Rad51-independent repair pathways.

**Conclusion**: Low-dosed Ganetespib significantly sensitizes cancer cells for proximal and distal SOBP proton irradiation. Ganetespib downregulates Rad51 protein and reduces the S/G2/M fraction of cells and is thus considered to suppress HRR. Hence, this study supports pursuing research on the combination of Ganetespib with proton radiotherapy for a prospective clinical exploitation.

## PTCNA-0079

### Mouse Lung FLASH Proton Radiation Using a 50 MeV Beam: Preliminary Results & Comparison to Conventional Dose Rate Proton Radiation

#### Ning Cao^1^, Sasha Tan^1^, Devin Miles^2^, David Argento^1^, Rob Emery^1^, Marissa Kranz^1^, Eric Ford^1^, Jing Zeng^1^

##### ^1^University of Washington, Department of Radiation Oncology, Seattle, USA ^2^Johns Hopkins University School of Medicine, Department of Radiation Oncology and Molecular Radiation Sciences, Baltimore, USA

**Purpose:** The normal tissue sparing effects of ultra-high dose rate radiation (FLASH) remain poorly understood. We present preliminary results of mouse FLASH proton radiation from a low-energy proton system (50 MeV) optimized for small animal radiobiological research.

**Methods:** We radiated 6-7 week old female C57BL/6 mice with whole lung radiation using the plateau region of a cyclotron-generated 50 MeV preclinical proton beam, transmitting through the whole mouse lung, with beam-shaping via customized vertical and horizontal collimators. Mice were stratified into 3 groups: 1) control/sham radiation; 2) conventional dose rate (17Gy at ∼0.5Gy/sec); and 3) FLASH (16-18Gy at 42-70Gy/sec). Mice were observed for dermatitis. Lung tissue was harvested post-radiation (1-hour, 5-days, 1-month, 3-months, 6-months). H&E and immunohistochemistry was performed for: yH2aX, cleaved caspase-3, and trichrome.

**Results:** Radiation dermatitis was different between FLASH and conventional groups: FLASH (grade 0-1=∼90%, grade 2=∼10%); conventional (grade 0-1=∼40%; grade 2-3=∼60%) [Figure 1]. One-hour post radiation, lower cleaved caspase-3 IHC staining was seen in the FLASH group versus conventional group, while yH2aX staining was similar in both groups [Figure 2]. More lung airspace disease (fluid and inflammatory cells) was seen in the conventional group at 6-months.

**Conclusion:** Preliminary results of mouse FLASH proton radiation from a 50 MeV beam suggest FLASH proton radiation leads to less normal tissue toxicity than conventional dose rate radiation. More studies are ongoing.

## PTCNA-0072

### A FLASH R&D proton beam line for advanced technology experiments and radiobiology research

#### Marta Rovituso^1^, Steven Habraken^2^, Mischa Hoogeman^2^, Marco van Vulpen^3^, Ellen Schenk^4^, Uli Weber^5^, Wouter van Burik^6^, Marco Lavagno^7^, Giuseppe Pittá^7^, Mattia Fontana^8^

##### ^1^HollandPTC, R&D, Delft, Netherlands ^2^HollandPTC, Medical physics, Delft, Netherlands ^3^HollandPTC, Medical doctor, Delft, Netherlands ^4^HollandPTC, Research and Development department, Delft, Netherlands ^5^GSI, Biophysics, Darmstadt, Germany ^6^HollandPTC, Research and Development, Delft, Netherlands ^7^DETECTOR, Research and Development, Turin, Italy ^8^Toret srl, Research and Development, Turin, Italy

**Introduction:** Ultrahigh dose rate (FLASH) proton therapy has the potential to better spare healthy tissues than proton therapy delivered at conventional dose rates. To safely test FLASH in clinical trials, many radiobiological and technological questions must be answered. Therefore, there is a need for an accessible and versatile R&D beam line to conduct FLASH experiments.

**Experimental setup:** The HollandPTC R&D room is equipped with a fixed horizontal beam line providing beam from 70 up to 240 MeV, and intensities from 1 to 800 nA. The room can provide single pencil beam and large fields with 98% beam uniformity and Spread-Out-Bragg Peak (SOBP) produced with 2D passive modulators. Recently, the maximum energy of 250MeV has been released in the R&D room for FLASH applications. The full beam characterisation has been performed together with absolute dose measurements.

**Results:** A 43% transmission efficiency of the ProBeam cyclotron is achieved at a 250 MeV energy. This resulted in a current of around 300 nA at target position. The beam spot size has a standard deviation of 3.6 mm. The fluence rate was found to be 8e6 protons/cm^2^s, more than a factor of 100 with respect to conventional beams. To further characterise the 250 MeV proton beam at maximum beam current a specific integral monitor chamber is currently under commissioning in collaboration with the company DE.TEC.TOR. Different cutting-edge solutions are adopted for the ionisation chambers to cope with FLASH intensities and minimise the recombination effects.The device is also equipped with X-Y strip ionisation chambers to measure beam size and position.

## Physics 1: Dosimetry and Treatment Planning PTCNA-0044

### Out-of-field dose in photon and hadron radiotherapy measured in a 3D-printed patient-specific anthropomorphic whole-body phantom

#### Hunter Tillery^1,2^, Phillip Taddei^3,4,5^, Meagan Moore^6^, Eric Leuro^4^, David Argento^5^, Gregory Moffitt^5^, Marissa Kranz^5^, Wayne Newhauser^7,8^, Kyle Gallagher^2^

##### ^1^McGill University, Medical Physics, Albany, USA ^2^Oregon Health and Science University, Radiation Medicine, Portland, USA ^3^Mayo Clinic, Radiation Oncology, Rochester, USA ^4^Seattle Cancer Care Alliance Proton Therapy Center, Radiation Oncology, Seattle, USA ^5^University of Washington School of Medicine, Radiation Oncology, Seattle, USA ^6^Louisiana State University, Biomedical Engineering, Baton Rouge, USA ^7^Louisiana State University, Physics and Astronomy, Baton Rouge, USA ^8^Mary Bird Perkins Cancer Center, Radiation Oncology, Baton Rouge, USA

**Purpose:** To compare out-of-field dosimetry in proton, neutron, and photon radiotherapy with a 3D printed anthropomorphic phantom created using a non-ionizing surface scan.

**Methods:** We used a 3D printed phantom and tissue-equivalent chamber to measure absorbed dose in a phantom constructed from surface imaging of a female volunteer. Absorbed dose was measured in locations approximating the isocenter, thyroid, pacemaker, esophagus, and fetus positions. Square intracranial fields ranging from 2.8cm^2^ to 12.8cm^2^ were delivered using 6 MV flattened and flattening-filter-free (FFF) photon therapy, magnetically scanned layered proton therapy, and 50.5 MeV proton generated fast neutron therapy.

**Results:** Among the radiotherapy modalities, proton therapy generated the smallest out-of-field absorbed dose. For both proton and photon therapy, locations far from the field edge absorbed dose was small but measurable with the exception of the esophagus and fetus positions in proton therapy for which the measured dose was not distinguishable from background. Compared to proton therapy, out-of-field dose produced by the 6 MV FFF photon therapy was 60% higher in the thyroid and 30% higher in the pacemaker. Out-of-field dose generated by FFF photon therapy produced less out-of-field dose than conventional photon fields. Out-of-field dose in neutron therapy was highest in all locations.

**Conclusion:** Our measurements demonstrated that out-of-field absorbed dose is reduced in magnetically scanned proton therapy more than photon therapy and is largest in neutron radiotherapy. In each modality distance from the field edge influenced the magnitude of the out-of-field absorbed dose.

## PTCNA-0091

### Impact of range uncertainty, setup errors, and breathing phase on dose-averaged linear energy transfer (LETd) distributions in PBS proton lung plan

#### Suresh Rana^1,2,3^, Erik Traneus^4^, Michael Jackson^5^, Linh Tran^3^, Anatoly Rosenfeld^3^

##### ^1^Oklahoma Proton Center, Medical Physics, Oklahoma City, USA ^2^Miami Cancer Institute- Baptist Health South Florida, Medical Physics, Miami, USA ^3^University of Wollongong, Centre for Medical Radiation Physics, Wollongong, Australia ^4^RaySerach Laboratories, Medical Physics, Stockholm, Sweden ^5^Prince of Wales Hospital, Radiation Oncology, Randwick, Australia

**Purpose**: The purpose of this study was to investigate the impact of range uncertainty in conjunction with setup errors on dose-averaged linear energy transfer (LET_d_) distribution in robustly optimized pencil beam scanning (PBS) proton lung plans. Additionally, the variability of LET_d_ distribution in different breathing phases of 4DCT data set was evaluated.

**Methods**: In this study, we utilized the 4DCT data set of an anonymized lung patient. The tumor motion was approximately 6 mm. A PBS lung plan was generated in RayStation using a robust optimization technique (range uncertainty: ±3.5% and setup errors: ±5 mm) on the CTV for a total dose of 7000 cGy(RBE) in 35 fractions. The average RBE was 1.1. The LET_d_ distributions were calculated for the nominal plan, 12 plan robustness scenarios (range uncertainty (±3.5%) in conjunction with setup errors (±5 mm)), and ten different breathing phases of 4DCT data set.

**Results:** For a nominal plan, the mean LET_d_ was— CTV: 2.22 keV/μm; heart: 5.94 keV/μm; normal lung: 3.40 keV/μm. For plan robustness scenarios, the mean LET_d_ was— CTV:2.26±0.22 keV/μm; heart: 5.88±0.50 keV/μm; normal lung: 3.40±0.28 keV/μm. For 10 breathing phases, the mean LET_d_ was— CTV: 2.22±0.04 keV/μm, heart: 5.89±0.17 keV/μm, and normal lung: 3.37±0.13 keV/μm.

**Conclusion**: The maximum difference in mean LET_d_ among plan robustness scenarios was higher than the one from ten different breathing phases. For our 4DCT data set, breathing motion has little effect on LET_d_ distribution in the CTV and organs at risk for PBS plan robustly optimized for the setup and range errors.

## PTCNA-0075

### Daily Imaging Surveillance and its Impact on Adaptive Replanning in IMPT of High-Risk Prostate with Pelvic Nodes

#### Zhong Su^1^, Randal Henderson^2^

##### ^1^University of Arkansas for Medical Sciences, Radiation Oncology, Little Rock, USA ^2^University if Florida Proton Therapy Institute, Radiation Oncology, Jacksonville, USA

**Introduction**: High-risk prostate cancer requires irradiation of both prostate and pelvic lymph-nodes. The inter- and intra-fraction prostate motion poses challenges for treating both targets. An image guidance surveillance program was implemented to evaluate the magnitudes of both inter- and intrafraction motion to identify adaptive replanning needs and to mitigate the impact of systematic errors during treatment.

**Materials and Method**: A total of 85 patients (all with implanted gold markers in the prostate) treated between January 2019 to January 2021 were selected for this study. Daily marker-based target alignment was performed for each patient. The relative position difference between marker and bony alignment were recorded and compared to the PTV margins of the pelvic-nodes. Patients with consistent prostate motion close to or exceeding pelvic node PTV margin expansion were identified and re-simulated/re-planned to mitigate the impact of systematic errors from interfraction prostate motion. Post-treatment orthogonal radiographs were also obtained to evaluate prostate intrafraction motion. Patients with intrafraction motion exceeds the prostate PTV margins were identified and replanned with larger PTV expansion.

**Results**: A total of 4 patients were identified for large systematic errors in interfraction prostate motion (Figure 1). After patients were re-CT simulated and replanned, the interfraction prostate motion error was largely removed. Four different patients were identified due to off-margin intrafraction motion magnitude (Figure 2) and were replanned with larger prostate PTV margins.

**Conclusion**: The IGRT surveillance program was implemented and identified 4 patients for re-simulation and re-planning due to large systematic interfraction prostate motion error; identified 4 different patients for re-planning due to large intrafraction error.

## PTCNA-0023

### Combined proton-photon treatments with a fixed proton beamline integrated into a conventional bunker for photon therapy

#### Silvia Fabiano^1^, Panagiotis Balermpas^1^, Matthias Guckenberger^1^, Jan Unkelbach^1^, Louise Marc^1^

##### ^1^University Hospital Zürich, Department of Radiation Oncology, Zurich, Switzerland

**Aim:** Proton therapy (PT) is still a limited resource mainly because current facilities are bulky and costly. We explore the potential of a new design for PT which may facilitate proton treatments in conventional bunkers and allow the widespread use of protons.

**Methods:** The treatment room consists of a Linac, a motorized couch for treatments in lying position, and a horizontal proton beamline equipped with beam scanning. When proton plans are suboptimal due to limitations in the beam directions, high-quality treatment plans may be obtained by delivering protons and photons in the same fraction. We demonstrate this concept for a nasopharyngeal cancer case. Treatment planning is performed by simultaneously optimizing IMRT and IMPT plans based on their cumulative physical dose. Stochastic optimization is applied to mitigate systematic setup and proton range uncertainties.

**Results:** The combined treatment uses photons to improve dose conformity while protons allow reducing the integral dose in normal tissues (Figure 1). The combined treatment improves on single-modality IMRT and IMPT plans for the main organs at risk (Figure 2a). The lower doses that can be obtained with combined treatment translate into a 10%, 6%, and 4% lower risk for oral mucositis, xerostomia, and dysphagia compared to the pure IMRT plan in the nominal scenario. Stochastic optimization yields robust plans although protons and photons deliver inhomogeneous dose contributions (Figure 2b).

**Conclusions:** Compact and affordable PT systems will likely include a fixed beamline rather than a gantry. When proton-only plans are suboptimal, proton-photon combinations may retain high treatment quality.

## PTCNA-0101

### Proton Therapy Treatment Planning For Spine Targets With Extensive High-Z Material Implants

#### Archana Gautam^1^, Atony Adair^2^, Falk Poenisch^1^, Catherine Evans^3^, Kevin Tran^3^, David Grosshans^4^, Thomas H Beckham^4^, Xiaorong Ronald Zhu^1^, Narayan Sahoo^1^

##### ^1^MD Anderson Cancer Center Proton Therapy Center, Radiation Physics, Houston, USA ^2^MD Anderson Cancer Center Proton Therapy Center, Proton Therapy Center, Houston, USA ^3^MD Anderson Cancer Center Proton Therapy Center, PTC Radiation Oncology, Houston, USA ^4^MD Anderson Cancer Center Proton Therapy Center, Radiation Oncology Department, Houston, USA

**Background:** This work describes our treatment planning strategies to reduce the effect of range and dose calculation uncertainties in the proton therapy plans for targets in the spine with high Z material implants.

**Method and Materials:** Treatment planning was carried out with Eclipse TPS from Varian Medical Systems. The CT numbers of the high Z-material and artifacts were overridden to the values corresponding to the proton RSP of the material and to surrounding tissue respectively. A posterior and two posterior oblique passively scattered proton fields were used to minimize the effect of dose calculation and range uncertainties. The accuracy of dose calculation in Eclipse was evaluated by recalculating the dose using Monte-Carlo simulation (MCS). The robustness of target coverage under 5 to 10 % range uncertainties was evaluated to ensure acceptable target coverage and sparing of organ at risk under worst case scenarios.

**Results:** The use of three fields was found to reduce the effect of range uncertainties and produces a more homogeneous dose distribution compared to that for single PA field. The 3D Max dose difference between calculated dose distributions from Eclipse and MCS for three field plans was found to be 4.1%.

**Conclusions:** Use of three fields is found to produce a proton therapy plan with acceptable dose distributions under dose calculation and range uncertainties. Even with the larger uncertainness due to the presence of high-Z material, the proton therapy plan was preferred over the photon plan due to normal tissue sparing.

## PTCNA-0076

### Evaluation of knowledge-based spot-scanning proton treatment planning model for prostate cancer patients

#### Casey Johnson^1^, Sheng Huang^1^, Chin-Cheng Chen^1^, Anna Zhai^1^, Haibo Lin^1^, Pingfang Tsai^1^

##### ^1^New York Proton Center, Physics, New York, USA

**Purpose**: To evaluate the knowledge-based model in prostate proton treatment planning.

**Methods**: The knowledge-based RapidPlan module in Varian Eclipse TPS (v16.1) was used for prostate proton treatment planning. A model was created and trained using 40 patients for a prescription of 70.2 Gy to the prostate and seminal vesicle in 26 fractions. The model was evaluated by analyzing the goodness-of-fit summary statistics and identifying possible outliers. The established model was then tested on five additional prostate patients. The model-generated plans were compared to clinical-used plans to determine the accuracy and performance of the model in target coverage and organs-at-risk (OAR) sparing.

**Results**: The chi-squared values of the Rapidplan training result were 1.077, 1.127, 1.208, 1.119 for bladder, left femur, right femur, and rectum, respectively. The average optimization time is less than 10 minutes in a single run. CTV D_99%_ was >99% for all RapidPlans. OAR sparing was superior with RapidPlan ΔD_Mean_= -1.12 Gy (bladder, 13.07 Gy vs. 14.20 Gy), ΔV_32_ = -0.61% (bladder, 19.07% vs. 19.67%), ΔD_Max_ = -7.50 Gy (Left femur, 22.6 Gy vs. 30.14 Gy), ΔD_Max_ = -7.8 Gy (Right femur, 22.43Gy vs. 30.26 Gy). Rectum V53.7Gy was superior in RapidPlan for 4 out of 5 patients, but the mean dose was worse overall (18.59 Gy vs. 16.32 Gy). Variation in the ΔD_Mean_ for the rectum was found ranged from 0.36 Gy to 8.13 Gy.

**Conclusion**: The RapidPlan model saved 66% of the planning time and produced comparable CTV coverage and superior or equivalent OAR sparing for prostate patients.

## PTCNA-0037

### Accuracy of proton range calculations with dual-layer CT: A Monte Carlo study with animal tissues

#### Vadim Moskvin^1^, Fakhriddin Pirlepesov^1^, Yue Yan^1^, Ozgur Ates^1^, William Myers^1^, Jinsoo Uh^1^, Li Zhao^1^, Yoad Yagil^2^, Thomas Merchant^1^, Chia-ho Hua^1^

##### ^1^St. Jude Children's Research Hospital, Department of Radiation Oncology, Memphis, USA ^2^Philips Medical Systems, Global Advanced Technology, Haifa, Israel

**Purpose:** To compare proton ranges calculated in the treatment planning system (TPS) based on images of dual-layer CT (DLCT) and single-energy CT (SECT) with those from Monte Carlo simulations.

**Methods and materials:** Electron density (ED), atomic number (Z), and conventional CT images were acquired on Philips IQon spectral CT for nine animal tissues in 19.5 cm × 9 cm × 19 cm acrylic boxes. The DLCT method directly utilized stopping power ratios (SPR) calculated from ED and Z. The SECT method mapped HU to SPR. The treatment plans of 150.3 MeV and 221.3 MeV pristine energy layers were individually created in Varian Eclipse for both methods. The SECT plan was simulated with a fully commissioned TOPAS-based Monte Carlo model of a synchrotron proton beam delivery system PROBEAT-V. The proton range, *R90*, determined by DLCT and SECT were comparatively evaluated against the MC model.

**Results:** For 221.3 MeV, the deviation of proton range in TPS from Monte Carlo simulation reduced from 1.9–4.6 mm for SECT to 0.2–1.3 mm with DLCT (Table 1). The largest deviations occurred in fresh and frozen lung tissues. For 150 MeV, differences were less dramatic. However, DLCT continued to agree better with Monte Carlo simulations than SECT for lung tissues.

**Conclusions:** The DLCT-based range calculation in TPS agreed with Monte Carlo simulation to within 1.3 mm of 30.96 cm water-equivalent length for all tested animal tissues and within 0.5 mm for lung tissues. This finding supports the adoption of DLCT for dose calculation in TPS.

## PTCNA-0102

### Study of usefulness of PTV in improving the robustness of IMPT plans for target coverage under range and setup uncertainties

#### Narayan Sahoo^1^, Xiaodong Zhang^1^, Yupeng Li^1^, Falk Poenisch^1^, Archana Gautam^1^, Thomas Whitaker^1^, Ming Yang^1^, Richard Wu^1^, Xiaorong Zhu^1^

##### ^1^UT MD Anderson Cancer Center, Department of Radiation Physics-Pt. Care, Houston, USA

**Objective:** This study aims to examine the usefulness of PTV created by a uniform expansion of CTV to improve the CTV coverage in robustly optimized intensity modulated proton therapy treatment plans.

**Method & Materials:** IMPT treatment plans were optimized in Eclipse TPS from Varian Medical Systems using Nonlinear Universal Proton Optimizer (NUPO) for targets in brain and other regions of head and neck. The CTV was selected for robust optimization under eight uncertainties scenarios corresponding 3.5% range uncertainties and +/-3 mm setup uncertainties. Two plans for each patient were created, one with and one without the non-robust minimum target coverage objective for PTV. The PTV was customized to avoid overlap with OARs. Robustness of the CTV coverage under the same eight uncertainties scenarios were analyzed for both plans. The D95 for the CTV in the uncertainties band DVH for the worst-case scenario was used to judge the usefulness of the PTV in improving the robustness of IMPT plans.

**Results:** The IMPT plans with the minimum PTV target coverage objective are found to have a better D95 for CTV compared to the IMPT plans without this objective in the plan optimization with comparable DVHs for the OARs.

**Conclusion:** The PTV was found to be useful in improving the robustness of the IMPT plans for target coverage under range and setup uncertainties.

## PTCNA-0059

### Assessment of fragment contributions to dose and estimates of relative biological effectiveness by common models in carbon radiotherapy

#### Shannon Hartzell^1^, Fada Guan^1^, Paige Taylor^1^, Christine Peterson^2^, Stephen Kry^1^

##### ^1^The University of Texas MD Anderson Cancer Center, Radiation Physics, Houston, USA ^2^The University of Texas MD Anderson Cancer Center, Biostatistics, Houston, USA

Several models of variable RBE may be implemented in clinical and research-based treatment planning systems for carbon radiotherapy, including the microdosimetric kinetic model (MKM), stochastic MKM (SMKM), and Local Effect Model I (LEM), which have not been thoroughly compared. This work compares how models handle carbon beam fragmentation, providing insight into where model differences arise. Geant4 Monte Carlo was used to simulate clinically realistic monoenergetic and SOBP carbon beams incident on a phantom. Using these, input parameters for each RBE model (microdosimetric spectra, double strand break yield, kinetic energy spectra, dose) were calculated for relevant fragment species (H, He, Li, Be, B, and C). Spectra for each fragment were used to calculate linear and quadratic portions of each RBE model, which were combined with reference values and physical dose to calculate RBE. Calculations found that secondary fragment contributions could exceed 20% of total physical dose (Figure). When calculated using identical beam parameters, RBE magnitude varied greatly across models and was typically lowest using MKM. When compared across fragments, RBE decreased with atomic number when Z<3 and increased when Z≥3 for RBE(MKM) and RBE(SMKM) (Table). RBE(LEM) increased with Z, until dropping sharply at Z=6. Trends of RBE by fragment varied by LET region for microdosimetric models only. This study demonstrated that secondary fragments can contribute notably to physical and estimated biological dose, indicating that fragmentation is an important factor in treatment delivery. Similar trends were seen in RBE fluctuations by atomic number for microdosimetric models, which differed from those of RBE(LEM).

## Clinics 1: Lung, Lymphoma, Breast, GI, GU, Gyne PTCNA-0085

### Comparison of proton versus photon radiation patient cohorts in a phase 2 trial of response-adaptive radiation in locally advanced NSCLC

#### Jing Zeng^1^, Daniel Hippe^2^, Hubert Vesselle^2^, Paul Kinahan^2^, Ramesh Rengan^1^, Stephen Bowen^3^

##### ^1^University of Washington, Radiation Oncology, Seattle, USA ^2^University of Washington, Radiology, Seattle, USA ^3^University of Washington, Radiation Oncology & Radiology, Seattle, USA

**Introduction**: For patients with locally advanced non-small cell lung cancer (LA-NSCLC), proton therapy can provide dosimetric advantages over photon radiation. However, it is unknown whether this translates into superior outcomes. We conducted a phase II clinical trial of chemoradiation for LA-NSCLC examining response-adaptive radiation dose-escalation and compared the proton and photon patient cohorts on this trial.

**Methods**: Forty-five patients with AJCCv7 stage IIB-IIIB NSCLC were prospectively enrolled (NCT02773238) in 2016-2019. All patients underwent chemoradiation (23=protons, 22=photon IMRT); 18 patients also received consolidation durvalumab. PET/CT was performed at week-3 during chemoradiation and response status was prospectively defined by multivariate changes in tumor-volume and metabolic-uptake. PET non-responders received 74Gy in 30 fractions whereas PET-responders received 60Gy in 30 fractions. Differences between cohorts were evaluated by Mann-Whitney U-tests and log-rank tests.

**Results**: The cohort of patients treated with proton radiation had significantly lower dose to the lungs and heart with similar PTV volume (Table 1). Median follow up was 19 months. There was no statistically significant difference in overall survival (2y 61% vs. 40%, p=0.10), progression-free survival (1y 66% vs. 43%, p=0.28), locoregional control (1y 89% vs. 78%, p=0.10), or pneumonitis rate (1y 35% vs. 45%, p=0.65) in the two patient cohorts (Figure 1).

**Conclusion:** Our cohort of patients treated with proton therapy had lower radiation dose to lungs and heart, although there was no significant difference in clinical outcomes. Our results maybe be limited by our sample size, and we await results from larger randomized trials.

## PTCNA-0024

### Evaluation of Pencil Beam Scanning and Double Scatter Proton Radiotherapy following Chemotherapy in the Treatment of Aggressive Mediastinal non-Hodgkin Lymphoma

#### Jonathan Baron^1^, Christopher M Wright^1^, Russell Maxwell^1^, Michele M Kim^1^, Michael J LaRiviere^1^, Amit Maity^1^, John P Plastaras^1^, Ima Paydar^1^

##### ^1^University of Pennsylvania, Radiation Oncology, Philadelphia, USA

**Introduction:** Multi-agent chemotherapy followed by proton radiotherapy (PRT) is a standard treatment option for aggressive mediastinal non-Hodgkin lymphoma (AMNHL). Long-term outcomes remain favorable and minimizing dose to organs at risk is paramount. We report outcomes of patients with AMNHL receiving chemotherapy followed by either pencil beam scanning (PBS) or double scattered (DS) PRT.

**Methods:** We retrospectively analyzed data from a convenience sample of 24 patients with AMNHL treated between 2012-2019 at our institution. Median age was 36 (range 21-74), and disease stage distribution was 10 stage I, 10 stage II, 2 stage III, and 2 stage IV. Patients received either R-CHOP (18/24) or R-CHP-BV (6/24). Bulky disease (≥7.5cm) was present in 22/24 and 10/24 had B symptoms. Radiation toxicity was graded by CTCAEv5.

**Results:** Median follow-up was 38.8 months (range 8.3-91.5 months) and median PRT dose was 30.6Gy (range 30-39.6Gy). DS was used in 13/24, PBS in 11/24, and both in 6/24. Deep inspiration breath hold (DIBH) was used in 10/24. Median mean lung dose was 4.6Gy (range 2.4-7.4Gy) for PBS and 6.8Gy (range 3-16.7Gy) for DS, while median mean heart dose was 10.5Gy (range 5.9-16.4Gy) for PBS and 7.3Gy (range .01-16.9Gy) for DS. Grade 1 (G1) toxicities occurred in 16 patients and G2 toxicities in 4. G1 radiation pneumonitis occurred in one patient with no ≥G2 pneumonitis. A complete metabolic response was observed in 87.5% of patients (Figure 1).

**Conclusion:** Consolidative PRT following chemotherapy for AMNHL resulted in favorable outcomes without high-grade toxicities.

## PTCNA-0057

### Systematic review of deep inspiration breath hold in proton therapy and IMRT for mediastinal lymphoma

#### Chirayu Patel^1^, Jennifer Peterson^2^, Marianne Aznar^3^, Yolanda Tseng^4^, Scott Lester^2^, Deanna Pafundi^5^, Stella Flampouri^6^, Bradford Hoppe^5^

##### ^1^MGH, Radiation Oncology, Boston, USA ^2^Mayo Clinic, Radiation Oncology, Rochester, USA ^3^University of Manchester, Division of Cancer Sciences, Manchester, United Kingdom ^4^University of Washington, Radiation Oncology, Seattle, USA ^5^Mayo Clinic, Radiation Oncology, Jacksonville, USA ^6^Emory, Radiation Oncology, Atlanta, USA

**Purpose:** To systematically review all dosimetric studies investigating the impact of deep inspiration breath hold (DIBH) compared with free breathing (FB) in mediastinal lymphoma patients treated with proton therapy as compared to IMRT-DIBH.

**Materials and Methods:** A systematic search in PubMed was done to identify studies of mediastinal lymphoma patients with dosimetric comparisons of proton-FB and/or proton-DIBH with IMRT-DIBH including mean heart, lung, and breast doses (named MHD, MLD, and MBD respectively). Case reports were excluded. As of December 2020, eight studies fit these criteria.

**Results:** The trends in dose are summarized in the table. MHD was reduced (n=2), similar (<1 Gy difference, n=2), or worse (2.5 Gy worse, n=1) for proton-FB compared with IMRT-DIBH. MLD and MBD in all studies were reduced for proton-FB compared with IMRT-DIBH. Proton-DIBH led to lower MHD (2.3-7.4 Gy difference,) and MLD (0.7-1.1 Gy difference) compared to proton-FB, while MBD remained within 0.3 Gy in all studies. Compared with IMRT-DIBH, proton-DIBH reduced the MHD (1.5-10.1 Gy, n=7) or was similar (n=1). MLD (1.7-3.9 Gy) and MBD (1.5-7.8 Gy) were reduced with proton-DIBH in all studies. Integral dose was similar between proton-FB and proton-DIBH, and both were substantially lower than IMRT-DIBH.

**Conclusion:** Accounting for heart, lung, breast, and integral dose, proton therapy (FB or DIBH) was superior to IMRT-DIBH. Proton-DIBH can lower dose to the lungs and heart even further compared with proton-FB. However, the substantial increase in physics resources required at simulation and treatment must be considered to ensure accurate reproducibility for proton-DIBH treatment delivery.

## PTCNA-0065

### Dosimetric impact of tissue expander rotation in breast cancer patients undergoing post-mastectomy intensity modulated proton therapy

#### Lindsay K Morris^1^, Trey C Mullikin^1^, Satomi Shiraishi^1^, Brent J Bachmann^1^, Ashley E Hunzeker^1^, Nicholas B Remmes^1^, Dean A Shumway^1^, Robert W Mutter^1^, Kimberly S Corbin^1^

##### ^1^Mayo Clinic, Department of Radiation Oncology, Rochester Minnesota, USA

**Purpose:** To report the dosimetric impact of rotation in temporary tissue expanders (TE) in patients receiving post-mastectomy intensity modulated proton radiotherapy (IMPT).

**Methods:** Between 2017-2020, we identified consecutive patients in an internal registry as having a rotated TE during treatment. TE rotations were identified on daily setup kV imaging or CT-scans. Clinical target volumes (CTV) and organs at risk (OAR) were contoured on post-rotation CT-scans. Analysis of pre- and post-rotation dosimetry was completed in ProKnow (Elekta, Stockholm, Sweden).

**Results:** Thirty-five patients with TE reconstruction undergoing IMPT were identified as having 47 instances of TE rotation and post-rotation CT-scans in treatment position available for analysis. 46/47 pre-rotation plans met CTV(range(r), 4005-5000cGy) coverage of D95%>95%, while 16/47 met this constraint post-rotation. All pre- and post-rotation plans met coverage of D90%>90%. 12/14 pre- and 7/14 post-rotation plans met a boost CTV(r, 5625-6000cGy) coverage of D90%>90%. D0.01cc[Gy] to the left anterior descending (LAD) artery increased 1.5-fold from an average of 13.6Gy to 19.6Gy. D0.01cc[Gy] to the right coronary artery (RCA) increased 1.4-fold from an average of 12.3Gy to 16.2Gy. LAD and RCA mean doses increased 2.4-fold and 1.5-fold, respectively. Mean heart dose increased 1.4-fold for right and left-sided plans, from an average of 0.88Gy to 1.19Gy for left and 0.50Gy to 0.68Gy for right-sided plans.

**Conclusions:** Tissue expanders can rotate during breast IMPT, potentially impacting both CTV coverage and dose to OARs. Awareness of the potential for TE rotation during daily imaging is warranted. Replan is usually indicated.

## PTCNA-0061

### Post-mastectomy intensity modulated proton therapy reduces skin dose compared with photon therapy

#### Kimberly Gergelis^1^, Arslan Afzal^1^, Trey Mullikin^1^, W Scott Harmsen^2^, Nicholas Remmes^1^, Satomi Shiraishi^1^, Sean Park^1^, Kimberly Corbin^1^, Robert Mutter^1^, Dean Shumway^1^

##### ^1^Mayo Clinic, Radiation Oncology, Rochester MN, USA ^2^Mayo Clinic, Biomedical Statistics and Informatics, Rochester MN, USA

**Purpose/Objective(s)**: Studies have suggested greater skin toxicity with post-mastectomy proton pencil-beam scanning radiotherapy (PRT) than photon radiotherapy (XRT). We aim to compare the target coverage and skin sparing of a large cohort of patients treated with post-mastectomy PRT and XRT at a tertiary cancer center.

**Materials/Methods:** Consecutive women with unilateral, non-inflammatory breast cancer treated with 50 Gy (RBE) to the chest wall and regional lymph nodes between 2015-2019 were included. PRT was administered with a median of two multi-field optimized fields (intensity modulated proton therapy). The chest wall skin was defined as the first 3 mm from the external body surface. PRT and XRT planning objectives with respect to skin were to achieve microscopic disease target coverage while limiting surface hot spots. For XRT 3-5 mm daily bolus was generally employed. PRT planning objectives for skin were V90%>90% and D1cc<105%.

**Results:** One hundred seventy-nine women were included, 96 receiving PRT and 83 receiving XRT (95% 3D conformal radiotherapy with a wide tangent technique, 5% intensity modulated radiotherapy). Bolus was utilized in 93% of XRT patients. There was no significant difference of clinical characteristics between the groups (Table 1). Clinical target volume coverage with 47.5 Gy was excellent with PRT and XRT. The median skin dose to 0.01 cc, 1 cc, and 10 cc were all significantly lower with PRT (Table 2).

**Conclusions:** Post-mastectomy PRT administered with our skin-sparing technique is associated with lower skin dose than XRT, while maintaining excellent target coverage.

## PTCNA-0100

### Proton beam therapy for large hepatocellular carcinomas in western patients

#### Matthew Greer^1^, Stephanie Schaub^1^, Avril O'Ryan-Blair^2^, Tony Wong^2^, Smith Apisarnthanarax^1^

##### ^1^University of Washington, Department of Radiation Oncology, Seattle, USA ^2^University of Washington, Seattle Proton Center, Seattle, USA

**Purpose/Objectives**: We present a single institution retrospective study on the clinical outcomes of Western patients with large hepatocellular carcinomas (HCCs) treated with proton beam therapy (PBT).

**Materials/Methods:** Fifty-one HCC patients with tumors ≥ 5 cm and ineligible for other liver-directed therapies were treated with PBT between 2014-2019 with a 15-fraction regimen of 45.0-67.5 Gy(RBE). Non-classic radiation-induced liver disease (ncRILD) was defined by a Child-Pugh (CP) score increase 2+ and/or RTOG grade 3 enzyme elevation. Overall survival (OS), progression-free survival (PFS), and local control (LC) were calculated using the Kaplan-Meier method and univariate predictors of OS by Cox regression analysis.

**Results**: Patients represented a high-risk cohort: 45% with BCLC stage C, 18% with CP-B/C cirrhosis, and a median gross tumor volume (GTV) diameter of 11.1 cm. Pencil beam scanning was used in 67% of patients. A simultaneous integrated boost technique was employed in 78% to achieve an average GTV mean BED of 87.0 Gy(RBE). Median follow-up for all patients was 10 months. 1-year OS and PFS were 57% (95% CI 42-70%) and 32% (95% CI 19-48%), respectively. 1-yr LC was 92% (95% CI 78-98%) with three isolated local failures. Out of field liver recurrences were the dominant pattern of failure. Six patients (14%) experienced ncRILD; all but one patient had baseline CP-B liver function.

**Conclusions:** In this largest series to date of Western HCC patients with high-risk large tumors, moderate dose escalated PBT results in excellent local control rates and acceptable toxicities. Out of field and distant failures remain problematic.

## PTCNA-0060

### Patient Reported Outcomes: Decreased Gastrointestinal Toxicity with Adjuvant Proton Beam Therapy Compared to IMRT for Uterine Cancer

#### Justin Anderson^1^, Molly Petersen^2^, Allison Garda^3^, Khaled Aziz^3^, Trey Mullikin^3^, Michael Haddock^3^, Ivy Petersen^3^, Sujay Vora^2^

##### ^1^Mayo Clinic Arizona, Radiation Oncology, Phoenix, USA ^2^Mayo Clinic Arizona, Department of Biostastistics, Phoenix, USA ^3^Mayo Clinic Rochester, Department of Radiation Oncology, Rochester, USA

**Purpose**: To compare PRO-CTCAE in patients with endometrial cancer receiving adjuvant pelvic radiotherapy with proton beam therapy (PBT) vs intensity modulated radiotherapy (IMRT).

**Materials and Methods**: Patients with uterine cancer treated with curative intent who received either adjuvant PBT or IMRT between 2014-2020 were identified. Patients were enrolled on a prospective registry using a gynecologic specific subset of PRO-CTCAE designed to assess symptom impact on daily living. Gastrointestinal questions included symptoms of diarrhea, flatulence, bowel incontinence, and constipation. Symptom based questions were on a 0-4-point scale, with Grade 3+ symptoms occurring frequently or almost always. Patient reported toxicity was analyzed at baseline, end of treatment (EOT), and at 3, 6, 9, and 12 months after treatment. Unequal variance t-tests were used to determine if treatment type was a significant factor in baseline adjusted PRO-CTCAE.

**Results**: Sixty-seven patients met inclusion criteria. Twenty-two received PBT and 45 patients received IMRT. Brachytherapy boost was delivered in 73% of patients. Median external beam dose was 45 Gy for both PBT and IMRT (range: 45-58.8 Gy). When comparing PRO-CTCAE, PBT was associated with less diarrhea at EOT (p=0.01) and at 12 months (p=0.24) compared to IMRT. Loss of bowel control at 12 months was more common in IMRT patients (p=0.15). Any patient reported Grade 3+ GI toxicity was noted more frequently with IMRT (31% vs 9%, p=0.09).

**Discussion**: Adjuvant PBT is a promising treatment for patients with uterine cancer and may reduce patient reported gastrointestinal toxicity compared to IMRT.

## PTCNA-0030

### Proton therapy for high risk prostate cancer: Results from the Proton Collaborative Group PCG 001-09 prospective registry trial

#### Shaakir Hasan^1^, Stanislav Lazarev^2^, Madhur Garg^3^, John Chang^4^, William Hartsell^5^, Henry Tsai^6^, Carlos Vargas^7^, Mark Mishra^8^, Charles B Simone^1^, Daniel Gorovets^9^

##### ^1^New York Proton Center, Radiation Oncology, New York, USA ^2^Mount Sinai Medical Center, Radiation Oncology, New York, USA ^3^Montefiore Medical Center, Radiation Oncology, New York, USA ^4^Oklahoma Proton Center, Radiation Oncology, Oklahoma City, USA ^5^Northwestern University, Radiation Oncology, Chicago, USA ^6^Procure New Jersey, Radiation Oncology, Somorset, USA ^7^Mayo Clinic, Radiation Oncology, Phoenix, USA ^8^University of Maryland, Radiation Oncology, Baltimore, USA ^9^Memorial Sloan Kettering Cancer Center, Radiation Oncology, New York, USA

**Introduction**: Using the Proton Collaborative Group (PCG) prospective registry, we report outcomes for high risk prostate cancer (HRPC) treated with proton therapy.

**Methods**: After exclusion, 605 HRPC patients from 8/2009-3/2019 at nine institutions were analyzed for freedom from progression (FFP), metastasis free survival (MFS), overall survival (OS), and toxicity. Multivariable cox/binomial regression models were used to assess for predictors of FFP and toxicity.

**Results**: [CS1] Median age was 71 years. Gleason grade groups 4 (49.4%) and 5 (31.7%) were most common, as were stage T1c (46.1%) and T2 (41.3%). The median pre-treatment prostate specific antigen was 9.18. Median dose was 79.2 GyE in 44 fractions. Pelvic lymph nodes were treated in 58.2% of cases and 63.6% of patients received androgen deprivation therapy. Pencil beam scanning was used in 54.5%, uniform scanning in 38.8%, and a rectal spacer in 14.2%. At a median follow-up of 2 years, the 3- and 5-year FFP were 90.7% and 81.4%, respectively. The 5-year MFS and OS were 92.8% and 95.9%, respectively. Independent correlates of FFP included Gleason ≥8, PSA >10, and cT2 (all P<0.05). There were no grade 4 or 5 adverse events. Late grade 2 and 3 genitourinary toxicity was 5.8% and 1.7%, respectively, while late grade 2 and 3 gastrointestinal toxicity was 5% and 0%. Grade 2 and 3 erectile dysfunction at 2 years was 48.4% and 8.4%, respectively.

**Conclusion**: In the largest series published to date, our results suggest that early safety and efficacy outcomes using proton therapy for HRPC are encouraging.

## PTCNA-0032

### Acute gastrointestinal (GI) and genitourinary (GU) toxicities of intensity-modulated proton therapy (IMPT) targeting prostate and pelvic nodes for prostate cancer

#### Chunhee Choo^1^, David Hillman^2^, Thomas Daniels^3^, Carlos Vargas^4^, Jean Claude Rwiigema^4^, Kimberly Corbin^1^, Bradley Stish^1^, William Wong^4^

##### ^1^Mayo Clinic, Radiation Oncology, Rochester, USA ^2^Mayo Clinic, Biomedical Statistics and Informatics, Rochester, USA ^3^NYU Langone, Radiation Oncology, New York, USA ^4^Mayo Clinic, Radiation Oncology, Scottsdale, USA

**Purpose**: To assess acute GI and GU toxicities of IMPT targeting prostate/seminal vesicles and pelvic lymph nodes for prostate cancer

**Methods**: A prospective study (ClinicalTrials.gov: NCT02874014) evaluating moderately hypo-fractionated IMPT for high-risk (HR) or unfavorable intermediate-risk (UIR) prostate cancer accrued 56 patients. Prostate/seminal vesicles and pelvic lymph nodes were treated simultaneously with 6750 and 4500 cGy RBE, respectively, in 25 daily fractions. All received androgen deprivation therapy. Acute GI and GU toxicities were prospectively assessed, using 7 GI and 9 GU categories of CTCAEv.4, at baseline, weekly during radiotherapy, and 3-month post-radiotherapy. Fisher exact tests were used for comparisons of categorical data.

**Results**: Median age: 75 years. Median follow-up: 25 months. 55 patients (52: HR; 3: UIR) were available for acute toxicity assessment. 62% and 2% experienced acute grade 1 and 2 GI toxicity, respectively. 65% and 35% had acute grade 1 and 2 GU toxicity, respectively. None had acute grade ≥ 3 GI or GU toxicity. The presence of baseline GI and GU symptoms was associated with a greater likelihood of experiencing acute GI and GU toxicity, respectively (Table 1 and 2). Of 45 patients with baseline GU symptoms, 44% experienced acute grade 2 GU toxicity, compared to only 10% among 10 with no baseline GU symptoms (p=0.07). Although acute grade 1 and 2 GI and GU toxicities were common during radiotherapy, most resolved at 3 months post-radiotherapy.

**Conclusions**: A moderately hypo-fractionated regimen of IMPT targeting prostate/seminal vesicles and pelvic lymph nodes yielded very acceptable acute GI and GU toxicity.

## Physics 2: Delivery Technics including FLASH and Spot-Scanning Proton Arc (SPARC) PTCNA-0038

### Assessing the interplay effect based on a precise machine-specific delivery sequence and time for cyclotron accelerator proton therapy system

#### Lewei Zhao^1^, Gang Liu^2^, Jiajian Shen^3^, Andrew Lee^4^, Di Yan^1^, Rohan Deraniyagala^1^, Craig Stevens^1^, Xiaoqiang Li^1^, Shikui Tang^4^, Xuanfeng Ding^1^

##### ^1^Beaumont Health System, Radiation Oncology, Royal Oak, USA ^2^Huazhong University of Science and Technology, Cancer Center, Wuhan, China ^3^Mayo Clinic Arizona, Radiation Oncology, Phoenix, USA ^4^Texas center for Proton Therapy, Radiation Oncology, Ivring, USA

**Purpose:** We proposed an experimental approach to build a precise machine-specific model for standard, volumetric, and layer repainting delivery based on a cyclotron accelerator system. Then, we assessed the interplay effect using a 4D mobile lung target phantom compared to a generic delivery sequence model from West German Proton Therapy Essen (WPE).

**Methods**: The machine delivery log files, from an IBA ProteusPLUS® system, were retrospectively analyzed to quantitatively model energy layer switching time, spot switching time, and spot drill time for standard and volumetric repainting delivery. To quantitatively evaluate the interplay effect, a series of digital thoracic 4DCT image sets were used. The interplay effect was assessed based on the 4D dynamic dose accumulation method. Different delivery technique such as standard delivery (n=1), volumetric repainting delivery (n=2,3,4) and layer repainting delivery (n=2,3,5,25) were simulated based on the machine-specific delivery sequence model and WPE model.

**Results:** The results showed that the WPE model's spot delivery sequence deviated from the log file significantly compared to the machine-specific model. Based on the treatment delivery calculation of a lung treatment plan with target size (65 mm^3^) and layer repainting 25 times (n=25), the difference is about 21.01%. Such a difference also resulted in different interplay effects estimation between the two models even though both institutions used the same proton system from IBA and calculated using the same 4DCT imaging set.

**Conclusion:** A precise machine-specific delivery sequence is highly recommended to ensure an accurate estimation of mobile target treatment's interplay effect.

## PTCNA-0056

### Assessment of shielding requirements for proton beam FLASH delivery

#### Francis Yu^1^, Minglei Kang^1^, Sheng Huang^1^, Chengyu Shi^1^, Chin-cheng Chen^1^, Shouyi Wei^1^, Qing Chen^1^, Jehee I. Choi^2^, Charles B. Simone II^2^, Haibo Lin^1^

##### ^1^New York Proton Center, Physics, New York, USA ^2^New York Proton Center, Radiation Oncology, New York, USA

**Purpose**: To evaluate the effectiveness of existing shielding in a dedicated research room (second floor) for proton FLASH beam delivery.

**Materials and Method**: The radiation survey was performed with Ludlum 42-38 WENDY-2 neutron detector and Ludlum 9DP ion chamber survey meter in a fixed horizontal beam room using an ultra-high dose rate proton beam (FLASH). A 250 MeV spot was delivered (total 5 minutes) with a cyclotron current of 600 nA (∼210 nA at the nozzle), which provided a spot peak dose rate of 805 Gy/s. The survey meters were moved around to identify the highest reading of each location, and the readers were compared to survey results of clinical standard-dose rate beams.

**Results**: The highest readings for the FLASH beam were along the beam path and read 550 μR/hour on WENDY-2 and 55 μR/hour on 9DP ion chamber meter. The neutron and photon readings are 97 to 170 fold higher than for clinical beams for the location with direct transmission. The readings are ∼28 fold higher in the control room due to the length of the maze. High activation of 650 mR/hour, 434 mR/hour, and 186 mR/hour was observed in the solid water beam stopper at isocenter 5, 30, and 60 minutes after FLASH delivery.

**Conclusion**: No extra shielding is needed to deliver FLASH beam in our research room. A beam-angle-dependent survey is recommended for the gantry room due to the flexible beam angles. Special attention should be paid to the activation of equipment in the treatment room.

## PTCNA-0066

### The First Modeling of the Spot-Scanning Proton Arc(SPArc) Delivery Sequence and Investigating Its Efficiency Improvement

#### Xuanfeng Ding^1^, Gang Liu^1^, Lewei Zhao^1^, Rohan Deraniyagala^1^, Craig Stevens^2^, Di Yan^2^, Xiaoqiang Li^1^

##### ^1^Beaumont Health, Radiation Oncology Proton Therapy Center, Royal Oak, USA ^2^Beaumont Health, Radiation Oncology, Royal Oak, USA

**Purpose:** Introduce an experimental approach to model a precise prototype arc system and quantitatively assess its efficiency improvement in the routine proton clinical operation.

**Methods:** The SPArc delivery sequence model(DSM_SPArc_) includes two kinds of parameters:(1) mechanical parameters. (2) irradiation parameters. Log files and an independent gantry inclinometer were used to derive the irradiation parameters through a series of test plans. The in-house DSM_SPArc_ was established by fitting both mechanical and irradiation parameters. Eight SPArc plans from different disease sites were used to validate the model's accuracy. To assess the treatment efficiency improvement, the DSM_SPArc_ was used to simulate the SPArc treatment delivery sequence and compared to the clinical IMPT treatment logfiles from the two full clinical days.

**Results:** The relative difference of treatment time between log files and DSM_SPArc_‘s prediction was 6.1%±3.9% on average, and the gantry angle vs. delivery time showed a good agreement between the DSM_SPArc_ and log file (Figure 1). Additionally, the SPArc plan could effectively save two hours out of ten hours of clinical operation by simplifying the treatment workflow for a single room proton therapy center. The average treatment delivery time (including gantry rotation and irradiation) per patient was reduced to 226±149s using SPArc compared to 665±407s using IMPT (p<0.01).

**Conclusion:** SPArc can offer a superior delivery efficiency to improve daily patient treatment throughput, compared to IMPT. Most importantly, this model helps the community to further develop and investigate this merging technqieu especially incorporating the arc delivery speed and time into the SPArc optimization algorithm.

## PTCNA-0048

### A direct machine-specific parameters incorporated Spot-scanning Proton Arc (SPArc) algorithm

#### Gang Liu^1^, Lewei Zhao^2^, Di Yan^2^, Xiaoqiang Li^2^, Xuanfeng Ding^2^

##### ^1^Beaumont Health System / Wuhan Union Hospital, Radiation Oncology, Royal oak, USA ^2^Beaumont Health System, Radiation Oncology, Royal Oak, USA

**Purpose:** To address the challenges of generating a deliverable and efficient spot-scanning proton arc (SPArc) plan for a proton therapy system. We developed a novel SPArc optimization algorithm (SPArcDMSP) by directly incorporating machine-specific parameters such as mechanical constraints and delivery sequence.

**Method and Material:** A SPArc delivery sequence model (DSMarc) was built based on the machine-specific parameters of the prototype arc delivery system IBA ProteusONE®. The SPArcDMSP resamples and adjusts each control point's delivery speed based on the DSMarc calculation through the iterative approach (Fig1). Users could set the expected delivery time and gantry max acceleration as a mechanical constraint during the optimization. Four cases (brain, liver, head neck, liver, and lung cancer) were selected to test SPArcDMSP. Two kinds of SPArc plans were generated using the same planning objective functions:(1) SPArcDMSP plan meeting the maximum allowable gantry acceleration speed(0.6deg/s2);(2) SPArcDMSP-user-speed plan with a user pre-defined delivery time and acceleration speed < 0.1deg/s^2. Arc delivery sequence such as gantry speed, delivery time was simulated based on the DSMarc and was compared.

**Results:** With a similar objective value, number of energy layers, and spots, both SPArcDMSP and SPArcDMSP-user-speed plans could be delivered continuously within the ±1 degree tolerance window.The SPArcDMSP-user-speed plan could minimize the gantry momentum change based on users' preference (Fig 2).

**Conclusions:** For the first time, the clinical users could generate a SPArc plan by directly optimize the arc treatment speed and momentum changes of the gantry. This work paved the roadmap for the clinical implementation of proton arc therapy in the treatment planning system.

## PTCNA-0080

### A quantitative dose perturbation comparison study between gold and platinum VISICOIL™ fiducial markers in proton beam therapy

#### Xuanfeng Ding^1^, Gang Liu^1^, Weili Zheng^1^, Jessica Valdes^1^, Xiaoqiang Li^1^, Lewei Zhao^1^, An Qin^1^, Daniel Krauss^1^, Di Yan^1^

##### ^1^Beaumont Health, Radiation Oncology Proton Therapy Center, Royal Oak, USA

**Purpose:** To quantitatively investigate the dose perturbations difference between gold and platinum VISICOIL™ fiducial markers in proton beam therapy

**Method:** Gold and platinum VISICOIL™ fiducial markers with two different dimensions were tested, including 0.35 & 0.5mm in diameter and 5mm in length. Total four kinds of markers. Gafchromic EBT2 was used to measure dose perturbations along the beam path in a “sandwich” setup (Figure 1). Dose perturbation was reported in each depth (0.3mm, 1.65mm, 3.00mm, 5.40mm, 7.80mm, 10.20mm, 12.60mm, 18.15mm). Relative proton stopping power relative to water were calculated through National Institute of Standards and Technology database and SRIM (version -2013) in the therapeutic energy range (70-220MeV).

**Result:** There is no statistical difference between gold and platinum VISICOIL™ fiducial markers in all the depth with diameter 0.35mm (p=0.125), and 0.5mm (p=0.130). The maximum point dose perturbation between Au and Pt marker with the same dimension are similar (0.35mm diameter at 7.8mm WET: 2.85%±2.31% Au vs. 2.70%±2.60% Pt; 0.5mm diameter at 5.4mm WET: 8.81%±2.60% Au vs. 8.81%±2.57% Pt.) (Figure 2). Bilateral treatment field arrangement could further reduce the dose perturbation by half. The relative stopping power ratio to water was calculated based on gold and platinum materials. The result showed there are about 3.5% difference between the two materials.

**Conclusion:** The study indicated that the Au and Pt VISICOIL™ fiducial markers have very similar physics properties and could interchangeable in the proton beam therapy as long as the clinical users correct the RSP during the planning process.

## PTCNA-0045

### Dosimetric impact of spinal implant on proton therapy plans for paraspinal target

#### Sheng Huang^1^, Pingfang Tsai^1^, Anna Zhai^1^, Arpit Chhabra^2^, Haibo Lin^1^, Chengyu Shi^1^

##### ^1^New York Proton Center, Medical Physics, New York, USA ^2^New York Proton Center, Radiation Oncology, New York, USA

**Purpose**: To evaluate the dosimetric impact of different spine implants on proton therapy for paraspinal targets using an in-house fast Monte Carlo dose calculation platform.

**Methods**: The commercial Eclipse TPS was used to generate proton plans for a representative spinal chordoma target in a spinal phantom with four different spine implants: normal tissue without an implant, titanium, carbon-fiber-reinforced polyetheretherketone (CFR-PEEK), and hybrid (CFR-PEEK screw with titanium head) implants. The in-house fast Monte Carlo dose calculation algorithm, MCsquare, was used to evaluate the impact of different implants on plan quality.

**Results**: Monte Carlo dose calculation revealed that up to a maximal 16% local dose shadow within target after titanium screw and rod, and it depends on the dimension of metal implant and also the beam arrangement. The D95 of the CTV50 [AC1] decreased by 8.2% and 4.5% for titanium and hybrid implant, respectively, but no meaningful difference was found for CFR-PEEK implant and normal spine when comparing TPS with MCsquare. Monte Carlo results show no impact on OARs' dosimetric merits.

**Conclusion**: Dose calculation accuracy of TPS is limited for scenarios with metal heterogeneity. Titanium implants, in certain circumstances, cause dose shadowing and could theoretically compromise target coverage. On contrary, the use of increasing CFR-PEEK proportion, especially with complete CFR-PEEK implants improves the overall dosimetry accuracy.

## PTCNA-0041

### Hip implant planning procedure for proton plans (HIPPPP)

#### Sean Boyer^1^, Linnae Campbell^1^, Steven Laub^1^, Mark Pankuch^1^, Maggie Stauffer^1^

##### ^1^Northwestern Medicine Proton Center, Physics & Dosimetry, Warrenville, USA

**Purpose:** While proton centers may observe a low frequency of prostate patients with titanium hip replacements, their plans require a specialized beam arrangement. Due to the increased complexity a subcommittee reviewed different treatment techniques to determine the optimal planning technique.

**Methods:** Plans were restricted to the Inclined Beam Line (IBL), where gantry angles must be 90 or 30 degrees from vertical. Plans were also created on non-hip-replacement CT datasets to increase the patient cohort.

Comparisons were made for:

- Three beam plans versus two
- Contra-lateral oblique angle versus Ipsi-lateral oblique
- Equally weighted beams versus increased lateral beam weight

Robustness testing using +/- 3.5% density shifts and +/- 3mm translational shifts (26 scenarios total) were performed for each plan. LET calculations were also done for each plan.

**Results:** There were minimal differences between all planning techniques.

The greatest differences:

- Three-beam plans had a higher DVH “low dose” region, two-beam plans had a higher “intermediate dose” region (Fig 1).
- The femoral head dose was slightly higher for plans with increased lateral weighting.

**Conclusion**: Since the different methods produced similar results, the priority then became which method was the most efficient for patient set up. The more efficient setup reduced treatment time and intrafraction motion. Therefore, the ideal beam arrangement for single hip-replacement patients would be two beams on the side opposite the hip implant with higher weighting of the lateral beam.

## PTCNA-0063

### Quantifying Voxel-Level Dose-Response Relationships for Non-Small Cell Lung Cancer Patients Treated with Protons and Photons

#### Yulun He^1^, Guillaume Cazoulat^1^, Carol Wu^2^, Carlos Cardenas^3^, Ezgi Kirimli^1^, Uwe Titt^3^, Julianne Pollard-Larkin^3^, Zhongxing Liao^4^, Radhe Mohan^3^, Kristy Brock^1^

##### ^1^MD Anderson Cancer Center, Imaging Physics, Houston, USA ^2^MD Anderson Cancer Center, Diagnostic Radiology, Houston, USA ^3^MD Anderson Cancer Center, Radiation Physics, Houston, USA ^4^MD Anderson Cancer Center, Radiation Oncology, Houston, USA

**Purpose**: To quantify voxel-level dose-response relationships for non-small cell lung cancer (NSCLC) patients treated with passive scattering proton therapy (PSPT) and intensity modulated radiation therapy (IMRT).

**Methods**: 203 locally advanced NSCLC patients treated on a prospective clinical trial with PSPT or IMRT were selected. For each patient, the planned dose was recomputed on the exhale planning 4DCT phase, and a 5-month post-treatment PETCT was obtained. Each planning/post-treatment CT-pair was registered via a biomechanical model-based deformable registration algorithm. Subsequently, voxel-level image density change (ΔHU) was calculated by subtracting the planning CT from the deformed PETCT to represent response. For each cohort, normalized mean ΔHU of voxels within the non-cancerous ipsilateral lung was fit to a standard Lyman NTCP model, and mean ΔHU of air voxels (<-800 HU) and tissue voxels (>-750 HU) in the planning CT was plotted against dose.

**Results**: Fifty-six patients with 74 Gy-RBE prescription dose have been analyzed to date. Figure 1 demonstrates a steeper NTCP curve for IMRT than PSPT.

**Conclusion:** The voxel-level dose-response relationship demonstrated a variable dependence on dose, supporting the hypothesis of higher linear energy transfer and relative biological effectiveness of low-dose protons compared to photons.

## PTCNA-0077

### A predictive maintenance approach for quality assurance of proton pencil beam scanning using unsupervised learning technique.

#### Tai Dou^1^, Nicolas Depauw^2^, Benjamin Clasie^2^, Tim Shen^2^, Jacob B. Flanz^2^, Kyungwook Jee^2^

##### ^1^Texas Center for Proton Therapy, Medical Physics, Irving, USA ^2^Massachusetts General Hospital- Harvard Medical School, Radiation Oncology, Boston, USA

**Introduction** Unscheduled machine downtime can cause patient treatment interruptions and may adversely impact patient treatment outcome. Conventional proton pencil beam scanning (PBS) quality assurance (QA) performs checks on proton beam parameters but does not reveal the underlying issues that the device may have prior to a machine failure. In this study, we propose a predictive maintenance approach that may provide early detection of machine issues.

**Methods** Log file data from daily morning QA performed at the Burr Proton Center of Massachusetts General Hospital were collected. Unsupervised deep learning-based model using Long Short-Term Memory Autoencoder (LSTM-AE) architecture was constructed. The model was trained on QA data of five “normal” sessions so that the model learns characteristics of normal machine properties. The model error (anomaly) is computed between the model predicted data and the measured data of the day and is converted to Mahalanobis distance (M-Distance) by comparing with a reference error distribution.

**Results** Figure 1. shows an overlay of model predicted M-Distance (blue) and downtime occurrences (red). Model prediction on the validation 2018 QA data shows that machine downtime events are associated with elevated peaks of M-Distance. Using an M-distance threshold of 22.09, our preliminary model prediction performance for three relevant machine anomaly event types is presented in terms of recall and precision rates in Table1.

**Conclusion** Our novel predictive modeling approach allows for the evaluation of abnormal machine status and demonstrates great promise for enabling predictive maintenance for proton PBS machines.

## Clinics 2: Practice, CNS, Pediatrics, Eye, Head & Neck PTCNA-0062

### Throughput simulation of a single-room proton therapy center using queuing theory

#### Richard Popple^1^, Rex Cardan^1^

##### ^1^The University of Alabama at Birmingham, Department of Radiation Oncology, Birmingham- AL, USA

**Purpose**: Beam time in a proton therapy center (PTC) is a scarce resource relative to demand. Understanding the capacity of a PTC is critical to patient, provider, and staff satisfaction, and to financial sustainability. Queuing theory is the mathematical framework for analyzing service-demand systems, such as patient flow through a clinic. We describe a model for simulating a single-room PTC.

**Methods**: The model is comprised of probability distributions for patient arrival, patient characteristics, and machine reliability. The distributions and parameters were selected to approximate the observed patient characteristics and machine maintenance records of a single-room PTC. Ten years of center operation at 16 hours per day were simulated for average patient arrival rates of 4-7.5 per week. The number of treatments delivered, machine availability, and patient wait times were recorded.

**Results**: Machine availability was 90% and the average time per treatment was 22.2 min. Box plots of number of treatments delivered per day and patient wait times versus patient arrival rate are shown below. The maximum capacity of the center was attained at 6.5 patients per week, resulting in an average of 35.3 treatments per day. Above 6.5 patients per week, wait times grow because patients arrive faster than treatments are completed.

**Conclusion**: We have demonstrated a throughput simulation of a single-room PTC. The model will be used to set realistic expectations for patient volume and to explore the effects of innovative operation strategies, such as selectively treating patients on weekends in anticipation of downtime events.

## PTCNA-0074

### Proton Therapy and the Radiation Oncology Alternative Payment Model

#### Aaron Bush^1^, Danushka Seneviratne^1^, Bradford Hoppe^1^, Mark Waddle^2^

##### ^1^Mayo Clinic, Radiation Oncology, Jacksonville, USA ^2^Mayo Clinic, Radiation Oncology, Rochester, USA

**Introduction**: The Centers for Medicare and Medicaid Services proposed Radiation Oncology Alternative Payment Model (RO-APM) intends to introduce an episode-based compensation model. We aimed to investigate the cost implications of proton therapy on this model.

**Methods**: We analyzed data on 259 patients who were treated definitively with protons from 2015-2016 at Mayo Clinic. Mean total radiotherapy costs were calculated using inflation adjusted standardized Medicare rates for all codes included in the RO-APM.

: Patients receiving protons composed of 8.7% of all radiotherapy patients. In contrast, national RO-APM data

**Results** estimated only 1.4% of patients would be treated with protons. Utilization of protons was most pronounced for upper GI(17.7%), CNS(15.4%), liver(11.9%), prostate(10.5%), head and neck(9.1%), and breast cancer(7.9%). This compared to RO-APM rates for upper GI(2.0%), CNS(2.2%), liver(3.0%), prostate(4.2%), head and neck(2.2%), and breast cancer(0.40%). Mean proton cost and percent difference compared to photons per disease site was: upper GI ($22,997; + 38.9% ), CNS ($28,427; + 52.2% ), liver ($18,299; + 69.3%), prostate ($25,843; + 7.4% ), head and neck ($28,330; + 14.5%), breast ($15,557; + 77.3%). Mean cost for all disease sites was $23,005 with protons vs. $14,765 with photons (T test; p < 0.01). Given a 7.3% utilization discrepancy and 55.8% mean cost increase, an adjustment of + 4.1% to national base rates would be required to accurately bundle compensation.

**Conclusions**: We propose that an adjustment factor accounting for the increased cost and utilization of proton therapy be implemented for future national base rate calculations.

## PTCNA-0094

### Quantitative evaluation of proton therapy related capillary leakage in glioma and meningioma using treatment response assessment maps (TRAMs)

#### Michaela Daniel^1^, Carola Lütgendorf-Caucig^1^, Gloria Zechmeister^2^, Markus Stock^3^, Leor Zach^4^, Orit Furman^5^, David Last^6^, Yael Mardor^6^, Eugen Hug^1^

##### ^1^MedAustron Ion Therapy Centre, Radiation Oncology, Wiener Neustadt, Austria ^2^Karl Landsteiner University of Health Sciences, Medicine, Krems, Austria ^3^MedAustron Ion Therapy Centre, Medical Physics, Wiener Neustadt, Austria ^4^Sheba Medical Center, Neuro-Oncology Unit- Radiation Oncology, Ramat Gan, Israel ^5^Sheba Medical Center, Neuro-Oncology translational research lab, Ramat Gan, Israel ^6^Sheba Medical Centre, The Advanced Technology Center, Ramat Gan, Israel

**Introduction**; One of the major effects of radiation is based on endothelial damage leading to an increased capillary leakage. MRI-based treatment response assessment maps (TRAMs) are established to qualitatively assess radiation induced capillary leakage based on contrast washout/accumulation over long delays. This is the first clinical study evaluating radiation induced small vessel damage during proton therapy (PT) both qualitatively and quantitatively.

**Materials and Methods**: Twenty-two patients (5 gliomas, 17 meningioma) were treated with PT. T1-weighted MR images were acquired 5 and 60 minutes post-contrast at five time points: before treatment (T1), mid-treatment (T2), end-treatment (T3), 6 months (T4) and 12 months follow-up (T5). TRAMs were generated by Sheba Medical Centre in Israel. Changes within tumours during radiation (T2, T3) and follow-up (T4, T5) compared to baseline (T1) were studied. The quantitative analysis was performed using MICE toolkit™ (Medical Interactive Creative Environment) software (Fig. 1) and included the % of GTV of each respective TRAM colour (red, blue).

**Results**: At baseline (T1) glioma GTVs presented on average 20±8% contrast clearance/AT compared to 78±4% in meningioma. Glioma GTVs showed non-significant decrease in clearance/AT during therapy which increased between end of therapy and follow-up; accumulation/TE decreased over all time points non-significantly. In meningioma GTVs contrast clearance/AT decreased significantly during therapy (T1-T3) and stabilized at follow-up (T4, T5).

**Conclusion**: Here we show for the first time radiation-induced changes in the tumour during and early after proton therapy based on TRAMs. Further evaluations and follow-up is needed to fully understand the clinical impact in terms of response-assessment.

## PTCNA-0054

### Clinical experiences in re-irradiation of recurrent meningioma

#### Ulrike Mock^1^, Carola Lütgendorf^1^, Birgit Flechl^1^, Maciej Pelak^1^, Markus Stock^1^, Antonio Carlino^1^, Marta Mumot^1^, Eugen Hug^1^, Piero Fossati^1^

##### ^1^MedAustron, Ion Therapy Center, Wiener Neustadt, Austria

**Introduction**: Excellent results have been reported for primary treatment of meningioma with RT. Few series have been published on re-irradiation following local failure.

**Materials and Methods**: Between 04/2017 and 01/2020, 21 patients with recurrent meningioma were re-irradiated using proton therapy. Initial treatment varied from single course of RT to multiple surgeries and irradiations. Patient- and treatment characteristics are summarized in Table 1.

**Results**: With a median follow-up of 22 months (range 7-42) local control (LC) at 2-years was 84%, and 2 years-overall survival (OS) was 87%. There was no acute toxicity ≥G3, but 2 late G3 toxicities: one patient with nasal synechiae (resolved after surgery) and one patient with CNS necrosis. 2-years-actuarial risk of persisting G3 toxicity was 4.8%. Sex and age had no correlation with outcome. Tumor grade correlated neither with OS nor with LC. At time of analysis, 100% Grade I meningioma were locally controlled versus 75% Grade II and 50% Grade 3. Time since last RT (> vs. < 5 years) correlated strongly with 2Y-OS (>5y 6 pts. 100% vs. <5y 15 pts 56%, p:019) and with 2Y-LC ( >5y 6 pts. 100% vs. <5y 15 pts 40% p .007). Re-irradiation dose (high>54 Gy 13 pts. vs low≤54 Gy RBE 8 pts.) showed a trend toward significance for 2-years LC (high-dose 74% vs. low-dose 100% p .08)

**Conclusion**: Proton re-irradiation is a safe and effective modality to successfully treat recurrent meningioma. The toxicity profile in our series was very favorable. Early recurrences after conventional RT have poor prognosis.

## PTCNA-0029

### Toxicity analysis of reirradiation with proton therapy for central nervous system tumors: a prospective proton collaborative group study

#### Shaakir Hasan^1^, Robert Press^1^, J Isabelle Choi^1^, George Laramore^2^, Henry Tsai^3^, Mark Mishra^4^, John Chang^5^, William Hartsell^6^, Charles B Simone^1^

##### ^1^New York Proton Center, Radiation Oncology, New York, USA ^2^University of Washington, Radiation Oncology, Seattle, USA ^3^ProCure New Jersey, Radiation Oncology, Somerset, USA ^4^University of Maryland, Radiation Oncology, Baltimore, USA ^5^Oklahoma Proton Center, Radiation Oncology, Oklahoma City, USA ^6^Northwestern University, Radiation Oncology, Chicago, USA

**Introduction**: This is an updated analysis of the acute toxicities of reirradiation with proton therapy (PT) for central nervous system (CNS) tumors using the Proton Collaborative Group (PCG).

**Methods**: The multi-institutional, prospectively collected PCG registry was queried for CNS tumors treated with PT reirradiation between 2010-2020. Acute grade 2 (G2) and grade 3 (G3) toxicities were reported, with binomial regression analysis to identify correlates thereof.

**Results**: Overall, 97 male and 79 female patients 19-85 years old (median 49) were identified, with 37 benign tumors, 117 gliomas, and 22 medulloblastoma/ependymomas/neuroendocrine tumors, located in cerebral hemispheres (n=130), infratentorium (n=24), base of the skull (n=14), and spinal cord (n=8). The median time to PT reirradiation was 63 months. Median PT dose and cumulative dose (EQD2) were 50 Gy_10_ (13 – 66 Gy) and 104 Gy_10_ (51-210 Gy_10_), respectively. Chemotherapy was given with PT in 86 patients. Baseline ECOG was 0 (n=55), 1 (n=56), 2+ (n=42). Median follow-up was 10 months. Acute G2 and G3 toxicities occurred in 51.1% and 7.9% of cases, respectively. Eighteen patients had G3 symptoms at baseline, and all but one resolved after PT. There were no grade 4 or 5 toxicities. Independent correlates of G3 toxicity per multivariable binomial regression analysis include ECOG 2 or higher (HR=18.7, P=0.003) and cumulative EQD2 dose over 115 Gy (HR=4.7, P =0.03).

**Conclusion**: Poor performance status and higher cumulative radiation doses in the salvage setting correlated with more G3 toxicity, but in appropriately selected patients reirradiation with PT for CNS tumors is well tolerated.

## PTCNA-0039

### Proton Plan Comparison Among Four Types of Spine Configurations

#### Chengyu Shi^1^, Haibo Lin^1^, Lei Hu^1^, Isabelle Choi^2^, Robert Press^2^, Shaakir Hasan^2^, Charles Simone^2^, Arpit Chhabra^2^

##### ^1^New York Proton Center, Medical Physics, New York, USA ^2^New York Proton Center, Radiation Oncology, New York, USA

**Purpose**: To evaluate a novel spine implant, carbon-fiber-reinforced polyetheretherketone (CFR-PEEK), for proton treatment planning.

**Methods**: We compared target coverage and sparing of organs at risk (OARs) for a spinal phantom with four different spine configurations: normal (no implant), titanium, CFR-PEEK, and hybrid (CFR-PEEK with titanium head). The spinal phantom was imaged via CT scan, and the iMAR CT set was used for planning. A representative spinal chordoma target and OARs were contoured. 50Gy was prescribed to the initial target volume, followed by a 24Gy boost, for which MFO proton plans were developed with 3 mm and 3.5% uncertainties. OAR dose constraints were set according to our institutional guidelines, including limiting the spinal cord Dmax <63Gy. We avoided any direct proton path through titanium parts per institutional practice.

**Results**: For the four spine configurations, the proton plans achieved similar nominal target coverage, heart mean, and spinal cord max dose. However, when evaluating coverage and OAR dose under uncertainty scenario analysis for initial CTV 50Gy 95% and 90% coverage, higher means and narrower range of doses were achieved for the normal and CFR-PEEK plans than the titanium and hybrid plans. Similarly, uncertainty analysis of spinal cord Dmax showed tighter distribution for normal and CFR-PEEK plans.

**Conclusion**: The CFR-PEEK implant has similar clinical properties to a normal spine for proton planning, allowing us to pass protons through the material and achieve superior target coverage and OAR sparing under nominal and uncertainty conditions as compared to treating in the presence of titanium hardware.

## PTCNA-0033

### Multi-Institutional Experience of Proton Therapy for Primary Central Nervous System Germinoma and Non-Germinomatous Germ Cell Tumors

#### Katherine Von Werne^1^, Haley Appel^1^, Rupesh Kotecha^1^, Muni Rubens^1^, Ralph Ermoian^2^, Henry Tsai^3^, Tamara Vern-Gross^4^, John Chang^5^, Stephen Mihalcik^6^, Matthew Hall^1^

##### ^1^Miami Cancer Institute, Radiation Oncology, Miami, USA ^2^University of Washington, Radiation Oncology, Seattle, USA ^3^Procure, Radiation Oncology, Somerset, USA ^4^Mayo Clinic, Radiation Oncology, Phoenix, USA ^5^Procure, Radiation Oncology, Oklahoma City, USA ^6^Northwestern University, Radiation Oncology, Chicago, USA

**Purpose:** To report on outcomes and toxicities following proton therapy (PT) for patients with primary central nervous system (CNS) germinoma and non-germinomatous germ cell tumors (NGGCT).

**Methods:** Data on patients with primary CNS germinoma and NGGCT treated with PT were queried from a prospective multi-institutional registry (PCG). We performed a similar query of our institutional database with IRB approval. Acute and late toxicities were scored using CTCAE v4.0.

**Results:** Forty-three patients (32 germinoma; 11 NGGCT) met the eligibility criteria, including 22 from PCG and 21 from our institution. Median age was 19 years (Range: 8-47). Twenty-three patients underwent surgery for tissue diagnosis and twenty were diagnosed based on imaging/laboratory values. Median PT dose was 36 Gy for germinoma patients (Range: 30-45 Gy); all NGGCT patients received 54 Gy. Twenty-three patients (22 germinoma, 1 NGGCT) received whole-ventricular irradiation and nineteen (9 germinoma, 10 NGGCT) received CSI. Median follow-up was 29 months (Range 2-101 months). At last follow-up, all had stable or controlled disease with 2-year disease-free and overall survival rates of 100%. Grade 2 alopecia was recorded in 33 patients (77%). Excluding alopecia, 15 patients (34%) developed any acute grade 2 non-hematologic toxicity, with only one grade 3 toxicity (fatigue). No late grade 2+ toxicities were reported.

**Conclusions:** In this multi-institutional study, patients treated with PT for CNS germinoma and NGGCT had high tumor control rates during early follow-up and few clinically significant acute or late treatment-related toxicities. Long-term outcomes for disease control and neurocognition are needed to measure the benefits of PT.

## PTCNA-0051

### Feasibility of pencil beam scanning proton therapy of ocular melanomas with a conventional gantry beam line

#### Sheng Huang^1^, Qing Chen^1^, Chengyu Shi^1^, Minglei Kang^1^, Christopher Barker^2^, Charles Simone^3^, Haibo Lin^1^

##### ^1^New York Proton Center, Medical Physics, New York, USA ^2^Memorial Sloan Kettering Cancer Center, Radiation Oncology, New York, USA ^3^New York Proton Center, Radiation Oncology, New York, USA

**Purpose**: To evaluate the feasibility of proton therapy of ocular melanomas using a non-dedicated treatment planning system (TPS) and proton pencil beam scanning gantry beam line.

**Methods**: The commercial Eclipse TPS was used to generate robust multifield optimized (rMFO) intensity-modulated proton plan for representative ocular tumor patients. Doses were compared among the initial plan and 40 additional scenarios of combined setup errors and range uncertainties. An in-house fast Monte Carlo dose calculation platform was used to assess the dosimetric impact of 3 tantalum fiducial markers for imaging-guidance treatment.

**Results**: Retina, optic nerve, cornea, lens, lacrimal gland, conjunctiva, sino-nasal mucosa and GTV were contoured on the treatment planning CT. 3-dimentional rMFO planning accounting for 2mm setup uncertainty and 3.5% range uncertainty was performed, utilizing 3 fields at different optimal gantry angles. All plans achieved satisfactory target coverage (TC), with at least 95% of CTV receiving full prescription of 50 Gy RBE in 5 fractions while achieving clinical dose limits of all organ at risks. The average target coverage remained D95=97.7% over 40 scenarios. Monte Carlo dose calculation revealed up to an 11% local dose shadow within target and D95 decreased by 3.2% if tantalum marker is in the beam path.

**Conclusion**: Non-dedicated TPS and gantry beam line can be used to effectively treat ocular tumors. This procedure is feasible with relatively low doses to anterior structures and achieves acceptable plan robustness. Fiducial markers could cause dose shadows and theoretically compromise local tumor control. Optimized beam angle and fiducial positioning should be considered.

## PTCNA-0036

### Contralateral Control Rate with Unilateral Proton Beam Radiotherapy for Oropharyngeal SCC: a multi-institutional prospective study from the Proton Collaborative Group

#### Aleks Vayntraub^1^, Thomas Quinn^1^, John Chang^2^, Noah Kalman^3^, Sanford Katz^4^, James Urbanic^5^, Arpi Thukral^6^, Jason Molitoris^7^, Craig Stevens^1^, Rohan Deraniyagala^1^

##### ^1^Beaumont Health System, Department of Radiation Oncology, Royal Oak- MI, USA ^2^Oklahoma Proton Center, Department of Radiation Oncology, Oklahoma City- OK, USA ^3^Baptist Health Proton Center, Department of Radiation Oncology, Miami- FL, USA ^4^Willis-Knighton Cancer Center, Department of Radiation Oncology, Shreveport- LA, USA ^5^University of California San Diego, UCSD California Protons, San Diego- CA, USA ^6^Northwestern University, Northwestern Proton Center, Warrenville- IL, USA ^7^University of Maryland, Maryland Proton Treatment Center, Baltimore- MD, USA

**Introduction:** Select patients with oropharyngeal SCC are candidates for unilateral radiation therapy. We sought to investigate if ipsilateral targeting leads to increased contralateral recurrences.

**Methods:** We queried the PCG database for patients treated with unilateral proton RT for head and neck SCC from 2015 – 2020 at 12 institutions. DICOMs were evaluated to ensure dose delivered matched a unilateral proton treatment plan. Demographic, clinical and pathological, toxicity and dosimetry information were compiled.

**Results:** We found 43 cases treated with unilateral proton RT. 94% (n=16) of recurrent cases received prior radiation. Oropharyngeal sites included tonsillar fossa (n=32), and base of tongue (n=11). 70% (n=30) of patients underwent concurrent chemotherapy - typically weekly cisplatin. The median dose and BED delivered was 69.96 CGE and 84.00 Gy respectively. Eight (18.6%) patients experienced at least one grade ≥3 toxicity. With a mean follow-up of 10.6 months (range 0 - 48) the local control rate at 1 year was 90.7%. All locoregional recurrences occurred within the ipsilateral neck; there were no contralateral failures. Distant metastasis developed in 4.6% of cases. For five cases (n=5), additional dosimetric analyses were performed for centralized review and revealed that ipsilateral level 2 doses were similar, whereas contralateral level 2 doses were higher with photons, mean: 15.4 Gy vs 0.36 CGE, D5%: 24.5 vs 4.62.

**Conclusions:** Unilateral Proton Beam RT for oropharynx cancer has similar disease control to photon therapy. The dosimetric advantage of proton beam therapy did not result in excess contralateral failures when compared to historical unilateral photon beam radiotherapy series.

## Multi-disciplinary: Carbon and Neutron PTCNA-0092

### Development of Intensity Modulated Neutron Therapy (IMNT) at the University of Washington (UW)

#### Robert D. Stewart^1^, George Laramore^1^, George Sandison^1^, Jonathan Jacky^2^, Matt Greer^1^, Natalie Viscariello^1^, David Argento^2^, Jing Zeng^1^, Jay Liao^1^, Upendra Parvathaneni^1^

##### ^1^University of Washington, Radiation Oncology, Seattle, USA ^2^University of Washington, Medical Cyclotron Facility, Seattle, USA

**Purpose:** High linear energy transfer (LET) neutrons have been used to treat over 3,300 patients at the UW because of their ability to overcome multiple mechanisms of resistance to low LET radiations. Technical and clinical challenges of implementing IMNT are presented along with an analysis of the potential therapeutic benefits.

**Methods:** A commercial treatment planning system (TPS) has been modified to incorporate neutron scattering kernels and accommodate the unique characteristics of the Clinical Neutron Therapy System (CNTS). A Monte Carlo model of the CNTS has been developed to independently confirm TPS doses. A portal imaging system based on ^11^C positron emission tomography has also been developed.

**Results:** Comparisons of measurements, TPS and Monte Carlo doses are in excellent agreement (3%/3mm g analysis) for a wide range of field sizes, both open and wedged. An analysis IMNT plans for seven head and neck patients shows an average 56% decrease in organ at risk dose compared to 3D conformal neutron therapy (3DCNT). The maximum dose decreased by 20% and 21% for the spinal cord and temporal lobe, respectively. The mean larynx D50% decreased by 80%. The overall number of monitor units for wedged and IMNT treatments is similar.

**Conclusions:** With IMNT, comparative planning studies demonstrate significant reductions in OAR dose are possible with similar target coverage. Clinical trials to compare 3DCNT to IMNT are in development. Such trials will inform ongoing work to evaluate the use of other types of high LET radiations for patient care, including carbon ions.

## PTCNA-0093

### Comparison of Micronuclei Formation by High LET Fast Neutrons and Low LET X-rays

#### Emily Hatch^1^, Devin Miles^2^, Ning Cao^3^, Peter Goff^3^, Robert Stewart^3^, Paul Nghiem^4^, Keith Stantz^5^, George A. Sandison^3^

##### ^1^Fred Hutchison Cancer Research Center, Basic Sciences and Human Biology, Seattle, USA ^2^Johns Hopkins Kimmel Cancer Center, Radiation Oncology, Seattle, USA ^3^University of Washington, Radiation Oncology, Seattle, USA ^4^University of Washington, Dermatology, Seattle, USA ^5^Purdue University, School of Health Sciences, Seattle, USA

**Purpose:** DNA fragmentation leads to micronuclei (MN) and release of self-DNA triggering the cGAS-STING pathway. Compared to low-LET radiation, we hypothesize that high-LET radiations 1) increase the number of MN per unit dose, and 2) rupture more frequently. Impact on MN for cells exposed to a DNA damage repair inhibitor (DDRi) were also assessed.

**Methods:** MN formation and rupture assessed in vitro using MCC-13 cells after 8 Gy of x-rays and 3 Gy of fast neutrons. Cells irradiated then fixed after first mitosis. Immunofluorescence markers were used for evaluation of DNA, MN rupture and plasma membrane integrity. Confocal microscopy imaging with automated image analysis provided: MN per cell, proportion of ruptured MN, and number of intact and ruptured MN. The proportion of ruptured MN was compared for x-rays and neutrons. Cells with ≥1 ruptured MN were scored at 38- and 72-hours post-irradiation. Additionally, cells were exposed to ATRi, a DDRi, for two hours pre-irradiation and MN analyzed at 72-hours.

**Results:** Per unit dose, high LET neutrons produced more MN than MV x-rays. The proportion of cells with at least one MN rupture at multiple time points was also greater for neutrons than x-rays. Exposure of cells to ATRi increased the MN number and the number of MN ruptures for both radiation types.

**Conclusions:** The RBE for double strand break (DSB) induction and RBE for MN induction are approximately the same. Fast neutrons may promote increased immunogenic cell death more efficiently than x-rays.

## PTCNA-0086

### Strategies and Challanges to Integrate Carbon Ions in Proton Therapy MedAustron - A Multi-Ion Therapy Center

#### Eugen B. Hug^1^, Markus Stock^2^, Antonio Carlino^2^, Piero Fossati^3^

##### ^1^EBG MedAustron GmbH, Management, Wiener Neustadt, Austria ^2^EBG MedAustron GmbH, Medical Physics, Wiener Neustadt, Austria ^3^EBG MedAustron GmbH, Carbon Ion Program, Wiener Neustadt, Austria

MedAustron began patient treatments with Proton Therapy in December 2016 and with Carbon Ion Radio-Therapy (CIRT) in July 2019. Currently, CIRT comprises 30% of Particle Therapy treatments and is either applied exclusively, or in combination as boost with Proton Therapy. All eligible patients participate in a prospective registry study. Figure 1 illustrates the distribution of all CIRT patients per indication and histology and figure 2 details the subgroup receiving combined Proton/CIRT. At the initial phase treatment selection was based on established CIRT indications, but rapidly expanded to take full advantage and explore the opportunities of Carbon Ion properties. Since CIRT was integrated into the pre-existing Proton Therapy program this presentation will focus on the clinical decision algorithm between Protons versus Carbon Ions or a combination of both. Principle factors involve radiobiologic considerations of possible improvement in local control for selected histologies and stages, physical advantages of Carbon Ions (sharp penumbra and small spot size), and optimal re-irradiation dose profiles and fractionation schemas. However, individualized risk assessment of particle therapy also takes into account the comparable large body of evidence on dose tolerance for Proton Therapy versus presently limited clinical data or extrapolated clinical normal organ tolerance data in case of CIRT. Examples will be presented. Optimization of Multi-Ion Therapy led to other innovative concepts, for example delivering high dose intra-tumoral CIRT boost without significantly increasing dose to normal tissues. CIRT was well tolerated and details of acute side effects on the initial 100 patients will be presented.

## PTCNA-0083

### Carbon-ion partial tumor irradiation targeting hypoxic segment and sparing the peritumoral immune microenvironment for unresectable bulky tumors: phase I trial.

#### Slavisa Tubin^1^, Piero Fossati^1^, Markus Stock^2^, Antonio Carlino^2^, Eugen Hug^1^

##### ^1^EBG MedAustron GmbH, Medical Department, Wiener Neustadt, Austria ^2^EBG MedAustron GmbH, Medical Physics Department, Wiener Neustadt, Austria

Extremely hypofractionated SBRT-based **PA**rtial **T**umor irradiation targeting **HY**poxic clonogenic cells (**PATHY**) but sparing peritumoral immune microenvironment (PIM) has previously been developed and clinically assessed for treatment of unresectable bulky, oligometastatic disease, showing encouraging results in terms of bystander and abscopal effects induction. Present study will be conducted to determine the immunogenic potential of carbon-ions applied to this novel concept. The hypothesis implies that for an effective immune modulation leading to improved therapeutic ratio, the entire tumor volume may not need to be irradiated but only a partial tumor volume, to initiate the immune cycle in radiation-spared PIM, resulting in tumoricidal bystander and abscopal effect. This is a mono-centric, prospective-phase I study which will enroll 23 patients with locally advanced or metastatic cancers with at least one bulky (≥6cm) lesion. This study uses a carbon-based PATHY approach, consisting of 3 consecutive 12Gy RBE fractions delivered exclusively to the hypoxic tumor segment while sparing PIM. The hypoxic segment will be defined using 64Cu-ATSM PET-CT and dynamic contrast enhanced MRT imaging. CARBON-PATHY will be administered at the precise timing, thus synchronized with the most reactive anti-tumor immune response phase based on the serially mapped homeostatic immune fluctuations by monitoring blood levels of the inflammatory markers. Primary endpoint will be bystander effect response rate defined as at least 30% regression of the unirradiated tumor tissue. Secondary endpoints will include overall survival, progression-free survival, abscopal response, symptoms relief, toxicity, feasibility of carbon-PATHY-timing and the bystander/abscopal response rate in relation to dose-size of PIM.

## PTCNA-0064

### Carbon Ion Radiotherapy for Treatment of Sacral Chordomas: An Institutional and National Comparison of Outcomes with Surgery and Primary Radiotherapy.

#### Elizabeth Jeans^1^, Yagiz Yolcu^2,3^, Jad Zreik^4^, Waseem Wahood^5^, Atiq ur Rehman Bhatti^2^, Mohamad Bydon^2,3^, Matthew Houdek^6^, Peter Rose^6^, Anita Mahajan^1^, Ivy Petersen^1^

##### ^1^Mayo Clinic, Department of Radiation Oncology, Rochester, USA ^2^Mayo Clinic, Neuro-informatics Laboratory, Rochester, USA ^3^Mayo Clinic, Department of Neurologic Surgery, Rochester, USA ^4^Central Michigan University, College of Medicine, Mount Pleasant, USA ^5^Dr. Kiran C. Patel, College of Allopathic Medicine, Davie, USA ^6^Mayo Clinic, Department of Orthopedic Surgery, Rochester, USA

**Purpose/Objectives:** Sacral chordomas are rare, locally aggressive neoplasms, for which both surgery and radiotherapy are utilized as local treatments. We compare outcomes for patients undergoing carbon ion radiotherapy (CIRT) versus surgical resection (SR) (+/- radiotherapy) versus definitive radiotherapy (DR) (proton/photon).

**Materials/Methods:** Propensity score matching was used to compare CIRT and SR from two institutional databases. Baseline characteristics, oncologic outcomes, functional mobility scale (FMS) and toxicities were compared.

Five subgroups from the National Cancer Database (NCDB), including patients treated with SR (with positive and negative margins) and DR (photon/proton), were matched to the CIRT cohort for outcomes analysis.

Cost of care within 2 years of treatment was analyzed.

**Results:** Forty-seven CIRT patients were matched to 47 SR patients with a median follow-up of 68.1 and 58.6 months, respectively. Baseline characteristics after matching were similar apart from poorer performance status in the CIRT cohort. After treatment, there was no difference in urinary retention, need for colostomy, overall survival (OS), progression-free survival, local recurrence, or distant metastasis between groups. Patients in the CIRT cohort had improved FMS and lower peripheral nerve toxicity (Table 1). In comparison of CIRT (n=188) to the NCDB subgroups (n=669), OS favored CIRT when compared to patients who had SR with a positive margin without adjuvant radiotherapy (p= 0.03, median follow-up 60.6 months). OS was similar between CIRT and DR proton patients and improved when compared to DR photon patients. Costs for CIRT and DR proton were lower than the SR cohort.

**Conclusion:** These data suggest CIRT is a safe, effective, and cost-effective treatment option.

## PTCNA-0034

### Accurate beam model and dose calculation algorithm for carbon ion radiotherapy under magnetic field toward adaptive particle radiotherapy

#### Takuto Miyoshi^1^, Shinichiro Fujitaka^1^, Taisuke Takayanagi^1^

##### ^1^Hitachi ltd., Research & Development, Ibaraki, Japan

This study aims at developing a pencil beam model for Magnetic Resonance Imaging guided Carbon Ion RadioTherapy (MRIgCIRT). The main issue was how to model the fragmentation of primary ^12^C ions and their magnetic deflection. Particles were classified into 3 groups according to the similarity of specific energy: group 1 (^12^C), group 2 (C isotopes other than ^12^C, B, Be and Li) and group 3 (other particles). In groups 1 and 2, the lateral distribution of physical dose was approximated by a Gaussian function, while the superposition of Gaussian and Lorentzian functions was used for group 3 to describe the halo which arises from light particles (Figure 1). The specific energy was considered to be constant at each depth. All parameters were obtained from Monte Carlo simulations using Geant4. To evaluate our model, biological dose distribution was calculated based on the micro dosimetric kinetic model and a lateral irradiation field was generated for comparison with the one simulated by Geant4. ^12^C beam was irradiated into a water phantom with 3-T magnetic field in the Geant4 simulation. Although the maximum of the absolute difference was increased for lower or higher energy, it did not exceed 2.7 % (Figure 2). This increase was probably due to overestimation by the Lorentzian function or the higher asymmetricity caused by beam deflection. The results of this work indicated that our model is valid for MRIgCIRT. Further research is needed to apply our model to a heterogeneous tissue.

## PTCNA-0015

### Estimating the need for carbon ion radiotherapy United States

#### Timothy Malouff^1^, Laura Vallow^1^, Danushka Seneviratne^1^, Anita Mahajan^2^, Robert Foote^2^, Bradford Hoppe^1^, Chris Beltran^1^, Steven Buskirk^1^, Sunil Krishnan^1^, Daniel Trifiletti^1^

##### ^1^Mayo Clinic, Radiation Oncology, Jacksonville, USA ^2^Mayo Clinic, Radiation Oncology, Rochester, USA

**Purpose:** Carbon ion radiotherapy (CIRT) is an emerging radiotherapy modality, although there are no centers in the US. We aim to estimate the need for a CIRT center in the United States.

**Materials and Methods:** Using the National Cancer Database, we analyzed the incidence of cancers treated with CIRT internationally (glioblastoma, hepatocellular carcinoma, cholangiocarcinoma, locally advanced pancreatic cancer, non-small cell lung cancer, localized prostate cancer, soft tissue sarcomas, and head and neck cancers) diagnosed in 2015. The percentage and number of patients likely benefiting from CIRT was estimated using inclusion criteria from clinical trials and retrospective studies, and this ratio was applied to 2019 statistics. An adaption correction rate was applied to estimate the potential number of patients treated with CIRT. Given the high dependency on prostate and lung cancers, the data were then re-analyzed excluding these diagnoses.

**Results:** Of the 1,127,455 new cases of cancer diagnosed in the United States in 2015, there were 213,073 patients eligible for treatment with CIRT based on inclusion criteria. When applying this rate and the adaption correction rate to the 2019 incidence data, an estimated 89,946 patients are eligible for CIRT. Excluding prostate and lung cancers, there were an estimated 8,922 patients eligible for CIRT. The need for CIRT is estimated to increase by 25- 27.7% by 2025.

**Conclusions:** Our analysis suggests a need for CIRT in the United States in 2019, with the number of patients possibly eligible to receive CIRT expected to increase over the coming 5-10 years.

## Poster Abstracts Biology: Enhanced biology in treatment planning PTCNA-0096

### Case Study: Evaluation of potential end-of-track effects in a base-of-tongue proton treatment following a severe and unexpected toxicity event

#### Alexander Egan^1^, Upendra Parvathaneni^1^, Jatinder Saini^1^, Tony Wong^1^, Robert D Stewart^1^

##### ^1^University of Washington, Radiation Oncology, Seattle, USA

**Purpose:** Despite contrary evidence in the literature, a constant proton relative biological effectiveness (RBE) of 1.1, regardless of linear energy transfer (LET), is still used for treatment plan evaluation and optimization. End-of-track effects (LET in excess of 5 keV / um) is an ongoing concern in proton therapy. We retrospectively analyzed the delivered treatment dosimetry and used published variable RBE models to evaluate the impact of high-LET track-ends in a patient with necrosis at the tongue base.

**Methods and Materials:**A 68-year old male with grade 4 necrosis of the soft tissue and hyoid bone approximately 3 months after definitive proton therapy for a T1N1 squamous cancer (concurrent chemotherapy). The region of gross disease received 69.96 Gy (RBE 1.1) in 33 fractions with pencil beam scanning. RBE-weighted dose (RWD) was evaluated using several variable (with LET) RBE models that span the range of possible RBE values at the track-end.

**Results:** Low-dose tissue regions adjacent to the tumor target are likely to have an elevated RBE (track-end effect). Variable RBE modeling suggests a 5-10% increase in the RBE-weighted dose (RWD) in the tissue regions with observed toxicity. We found no evidence the delivered treatment differed from the planned treatment (RBE = 1.1).

**Conclusions:** The unexpected treatment toxicity observed in this patient cannot be easily explained from a dosimetric perspective (RBE = 1.1) or in terms of the RWD computed using several, published variable RBE models. Other patient-specific factors likely contributed to the observed clinical outcome.

## Clinics: CNS PTCNA-0040

### Proton (IMPT) vs. Photon (VMAT) Planning Study for Spinal Chordoma

#### Chengyu Shi^1^, Haibo Lin^1^, Lei Hu^1^, Isabelle Choi^2^, Robert Press^2^, Shaakir Hasan^2^, Charles Simone^2^, Arpit Chhabra^2^

##### ^1^New York Proton Center, Medical Physics, New York, USA ^2^New York Proton Center, Radiation Oncology, New York, USA

**Purpose**: To compare IMPT vs. VMAT treatment plans for a spinal chordoma tumor using four unique spine configurations.

**Methods**: A representative 14 cm mid-thoracic chordoma was simulated in a spine phantom using four unique spine configurations: 1). normal (no spine) implant, 2). titanium, 3). a novel carbon-fiber-reinforced polyetheretherketone (CFR-PEEK) implant, and 4). hybrid implant (CFR-PEEK screw with titanium head). A sequential plan delivering 50Gy to the initial target volume followed by a 24Gy boost was prescribed in the four configurations (8 plans for proton and 8 plans for photon). MFO-IMPT technique was used for proton planning, whereas VMAT was used for photon planning. Organs at risk (OAR) dose constraints were set according to our institution guidelines, including spinal cord D_max_ <63Gy. Dose parameters of D_90%_, D_95%_, D_max_, D_mean_ were collected for the targets and OARs.

**Results**: No significant differences in target coverage were present between proton and photon plans (p=0.344, 0.093, 0.680, 0.311 for 1)-4) spine configurations considering 95% target coverage). Proton plans achieved a lower mean heart, mean left lung, and mean right lung doses, as well as reduced maximum spinal cord and esophageal doses. The proton plans, however, had a higher maximum skin dose.

**Conclusion**: Proton and photon planning can achieve similar target coverage for both the native spine and in the presence of spinal hardware. However, proton plans in all tested spine configurations achieve superior normal tissue sparing, with the exception of skin dose.

## PTCNA-0081

### Patients with meningioma I° and involvement of the optical structures: does proton therapy lead to patient-reported changes in vision?

#### Birgit Flechl^1^, Lisa Konrath^1^, Eugen B. Hug^1^, Carola Lütgendorf-Caucig^1^, Achtaewa Milana^1^, Pelak Maciej^1^, Annemarie Schallerbauer-Peter^1^, Jana Zimmermann^1^, Marion Sebek^1^, Petra Georg^1^

##### ^1^EBG MedAustron GmbH, Medical Department, Wiener Neustadt, Austria

**Objective**: Patients undergoing radiotherapy for meningiomas in close proximity to optic structures require particular attention towards maintaining visual status. This study reports on the patient-reported, prospective assessment of the visual performance following proton therapy (PT).

**Methods**: All patients treated with PT for meningioma WHO I, whose planning target volumes included parts of the optic system, were included. Assessment tool was the Visual Disorder Scale (VDS), of the EORTC-BN20 questionnaire. Test times were at start of PT, at completion, and at 3, 6, 12 and 24 months (mo) of follow-up (FU,t1-t6). A minimum FU of 6mo was required.

**Results**: With a mean FU period of 23.6mo 56 patients, aged 24-82 years (mean=53.9), received the institutional prescription dose of 54.0 GyRBE at 2.0 dose/fraction. The mean/D2% doses for optic chiasm and ipsilateral optic nerve were 43.4 GyRBE/49.9 GyRBE and 35.6 GyRBE/51.7 GyRBE, respectively. Mean/D2% doses for the contralateral optic nerve were 18.8 GyRBE/42.4 GyRBE. 302 data sets were analyzed (t1/t2/t3/t4/t5/t6: n=56/56/48/56/52/34). The mean symptom burden largely decreased over time (graph 1). At 12mo-FU, the subjective visual performance improved significantly (*p*=0.041), 13/15 asymptomatic patients reported no new onset of symptoms, 34/37 symptomatic patients experienced stabilization/improvement and 3/37 reported graduate worsening. Objective eye tests available on 21/52 patients confirmed the trend towards improvement in visual acuity.

**Conclusion**: Proton therapy of patients with meningioma WHO I in close proximity to optical structures provides excellent prospect of maintaining visual status. At 12mo-FU there was a statistically significant improvement in the perceived visual performance.

## Clinics: Pediatrics PTCNA-0058

### Seattle proton anesthesia reduction initiative (SPARI): employing checklists to maximize the number of pediatric patients safely treated awake

#### Molly Blau^1^, Stephanie Schaub^1^, Sally Rampersad^2^, Ralph Ermoian^1^

##### ^1^University of Washington, Radiation Oncology, Seattle, USA ^2^Seattle Children's Hospital, Anesthesiology- Pain Medicine, Seattle, USA

Daily anesthesia for pediatric patients undergoing proton therapy (PT) has potential to increase neurocognitive adverse treatment effects. It is emotionally and logistically difficult for patients and families, with NPO requirements exacerbating nutritional challenges and necessitating longer time in the center. Daily anesthesia also demands more health care resources including anesthesiologists, nursing support, increasing CT simulation and treatment time, and limiting scheduling flexibility for other patients. We aimed to develop a new tool for identifying and addressing barriers to children ≥ 3 years old completing PT awake. Checklists are commonly employed in radiation oncology and anesthesia, but have not been described in this context. We are not aware of prior research examining how strategies are implemented to avoid anesthesia, nor assessing residual barriers to treatment awake in patients continuing to require anesthesia. We developed checklists to be completed by the Radiation Oncologist and Anesthesiologist at simulation and weekly throughout treatment. These prompt the clinician to use several anesthesia-avoiding strategies, outlined in Table 1, and to document the remaining barriers to the patient being treated awake, shown in Figure 1. As part of an IRB approved quality assurance study, we will analyze data collected from these checklists. Through this rigorous method of implementing anesthesia-avoiding strategies, we expect to reduce anesthesia use and its impacts for children undergoing proton therapy. Equally important, we expect to describe which interventions are effective at what stage in the treatment course and to identify persistent barriers in patients who continue require anesthesia.

## Clinics: Breast PTCNA-0103

### Outcomes of Adolescents and Young Adults Following Radiotherapy for Breast Cancer

#### Danielle Cunningham ^1^

##### ^1^Mayo Clinic, Radiation Oncology, Rochester, USA

**Purpose:** Adolescent and young adult (AYA) with cancer face unique challenges.

**Methodology:** Retrospective review of AYA patients (age 15-25) treated with breast RT.

**Results:** Eleven AYA patients with breast cancer were treated with RT from 1998-2020; eight received RT. With 10-year median follow-up 88% are alive without disease. One died of metastatic disease and one had in-breast recurrence at 21 years. Median age at diagnosis was 24. All presented with palpable mass. Seven were invasive ductal carcinoma, one was adenoid cystic carcinoma. One was pregnancy associated. All underwent genetic testing, and 2 had BRCA1 mutations. All saw a fertility specialist, 4 elected oocyte retrieval (2) or leuprolide (2). Stage ranged from I-IIIC (Stage I (1), II (3), III (4)). Five were ER+/PR+/HER2-, 1 was triple positive, and 2 were triple negative. Two underwent lumpectomy, 6 had mastectomy, and 3 had contralateral prophylactic mastectomy. Four underwent reconstruction. Six had chemotherapy. 6 were treated with comprehensive post mastectomy RT and 2 had breast only RT. Proton therapy was used in 3. All experienced acute grade 1-2 dermatitis, no grade 3 or higher toxicities. Three developed grade 2 arm lymphedema at a median of 9 mo post RT, each had axillary dissection. Three developed shoulder dysfunction at a median of 10.9 mo after RT.

**Conclusions:** AYA patients with breast cancer have unique challenges. Among those undergoing RT, oncologic outcomes appear excellent. Extremity lymphedema and shoulder dysfunction were common.

## Clinics: Lung PTCNA-0087

### Safety and efficacy of ablative proton therapy for thoracic tumors

#### Kaitlin Qualls^1^, Trey C Mullikin^1^, Feven Abraha^2^, William Breen^1^, Danielle Cunningham^1^, Yolanda Garces^1^, Kenneth R Olivier^1^, Sean Park^1^, Kenneth Merrell^1^, Dawn Owen^1^

##### ^1^Mayo Clinic, Radiation Oncology, Rochester- MN, USA ^2^Mayo Clinic, Biostatistics, Rochester- MN, USA

**Purpose**: To report our initial experience using ablative dose intensity modulated proton therapy (IMPT) for lung lesions.

**Methodology:** Local, regional, and distant progression and overall survival (OS) were assessed in 34 patients who received ablative (BED10 > 70) IMPT from 2017-2020.

**Results**: Patient and treatment characteristics can be seen in Table 1. With median follow up of 14 months, 29 patients had partial or complete response as their best treatment response, and 4 had stable disease on post-treatment imaging. OS at 1 and 2 years were 64.9% and 48.2%, respectively, with median OS of 16.1 months. Six patients developed local recurrence (LR). Cumulative incidence (CI) of LR was 10.9% at 1 year and 26.1% at 2 years. Ten patients had a regional recurrence, with a CI of 21.9% at 1 year and 31.3% at 2 years. Seventeen developed distant progression, with a CI of 46.5% and 57.7% at 1 and 2 years. Univariate analysis did not identify any factors associated with increased risk of LR.

Acute grade 2+ toxicity was seen in 2 patients who developed dyspnea. Subacute grade 2+ toxicities occurred in 4 patients: 2 with radiation pneumonitis (grade-2), 1 with bronchial stenosis (grade-2), and 1 with bronchial obstruction (grade-3). The patient with bronchial obstruction also had a trapped lung (grade-4) requiring surgical management. Both pneumonitis patients had prior ipsilateral lung radiation.

**Conclusions:** Ablative IMPT provided favorable oncologic outcomes with a low rate of toxicity and should be considered especially for patients with underlying ILD or prior lung radiation.

## Clinics: GI PTCNA-0049

### Comparison of demographics and acute clinical events in pancreatic cancer patients treated with IMRT vs. Protons

#### Danushka Seneviratne^1^, Stephen Ko^1^, Christopher Hallemeier^2^, Jonathan Ashman^3^, Laura Vallow^1^, Mark Waddle^2^, Sunil Krishnan^1^

##### ^1^Mayo Clinic, Radiation Oncology, Jacksonville, USA ^2^Mayo Clinic, Radiation Oncology, Rochester, USA ^3^Mayo Clinic, Radiation Oncology, Phoenix, USA

**Introduction**: There are limited studies comparing the acute toxicities of protons vs. intensity modulated radiotherapy (IMRT) in the local treatment of pancreatic cancer. The objective of this study was to compare patient demographics and the incidence of acute clinical events in pancreatic cancer patients treated with IMRT vs. protons.

**Methods**: We collected data on 98 pancreatic cancer patients who were treated with IMRT or protons between 2007-2017 at the three Mayo Clinic locations.

**Results**: Patient characteristics are shown in Table 1. We found that mean age, gender, stage, and chemotherapy use were well balanced among the two treatment modalities. Interestingly, surgical management of the two groups differed significantly. Acute clinical incidents occurring within 90 days of radiation (blood transfusions, weight loss of >10%, emergency department visits, inpatient admissions, narcotic use, and death) did not significantly differ between IMRT vs. protons (Chi Square; p >0.05). Both treatments led to a significant reduction in the lymphocyte count from the start to the end of treatment. (T-Test; p < 0.05). The absolute value of this lymphocyte count drop during radiation therapy was similar between IMRT and protons. The total healthcare cost (comprising of radiation, chemotherapy, hospitalizations, ED visits, procedures etc.) was similar between the two modalities (T-Test; p > 0.05).

**Conclusions**: Our data indicate that both protons and IMRT are appropriate treatment modalities, with similar rates of acute events and total healthcare costs. Surgical management was higher among those treated with protons; however, this may reflect a selection bias for healthier patients undergoing proton treatments.

## PTCNA-0069

### Esophageal chemoradiation utilizing a single posterior proton beam technique with pencil beam scanning: Feasibility, dosimetry, and clinical outcomes

#### Tyler Gutschenritter^1^, Amber Post^1,2^, Steven R. Bowen^1,3^, Bao-Ngoc Thi Nguyen^4^, Veena Shankaran^5^, David B. Zhen^5^, Farhood Farjah^6^, Brant K. Oelschlager^7^, Jing Zeng^1^, Smith Apisarnthanarax^1^

##### ^1^University of Washington, Radiation Oncology, Seattle, USA ^2^Virginia Mason, Radiation Oncology, Seattle, USA ^3^University of Washington, Radiology, Seattle, USA ^4^SCCA Proton Therapy Center, Dosimetry, Seattle, USA ^5^University of Washington, Medical Oncology, Seattle, USA ^6^University of Washington, Cardiothoracic Surgery, Seattle, USA ^7^University of Washington, General Surgery, Seattle, USA

**Background**: Retrospective analyses and a phase II trial have demonstrated that chemoradiation with proton therapy in locally advanced esophageal cancer may reduce treatment-related toxicity, such as lymphopenia and cardiopulmonary complications. An unanswered question is the optimal proton beam arrangement to achieve the maximal reduction in radiation dose to both the heart and lung.

**Methods**: Retrospective review was performed of patients with locally advanced, non-metastatic esophageal cancer treated with chemoradiation utilizing a single posterior-anterior (PA) proton beam technique with pencil-beam scanning (PBS) at a single institution between January 2015 and August 2020. Inclusion criteria: 1) planned with pencil beam scanning (PBS) to 50.4 Gy(RBE) over 28 fractions, 2) concurrent carboplatin and paclitaxel, and 3) age 18 or older.

**Results**: Fifty patients met inclusion criteria of which 42 received trimodality therapy. Median follow-up was 3.2 years. 3-year overall survival (OS) and disease-free survival (DFS) were 77% and 56%, respectively. Nine patients (18%) experienced late grade 3+ toxicity, all of which were non-malignant esophageal strictures. No vertebral body fractures were observed. On univariate analysis, both ypN stage (HR 2.54 [1.04-6.25], p=0.04) and PTV volume (HR 1.68 [1.04-2.73], p=0.03) were significantly associated with DFS. No dosimetry variable was significantly associated with OS or toxicity. Of those undergoing esophagectomy (n=42), pCR was achieved in 16.7% (n=7).

**Conclusions**: Chemoradiation utilizing a single PA proton beam with PBS for locally advanced esophageal cancer is feasible and safe with clinical outcomes comparable to historic data.

## PTCNA-0082

### Proton therapy for unresectable and medically inoperable pancreatic cancer: multi-institutional prospective results from the proton collaborative group

#### Arpit Chhabra^1^, John Chang^2^, Michael Durci^3^, Nasiruddin Mohammed^4^, Henry Tsai^5^, Smith Apisarnthanarax^6^, William Regine^7^, Carlos Vargas^8^, Romaine Nichols^9^, Charles Simone II^1^

##### ^1^New York Proton Center, Department of Radiation Oncology, New York, USA ^2^The Oklahoma Proton Center, Department of Radiation Oncology, Oklahoma City, USA ^3^Willis Knighton Cancer Center, Department of Radiation Oncology, Shreveport, USA ^4^Northwestern Medicine Proton Center, Department of Radiation Oncology, Warrenville, USA ^5^Procure Proton Therapy Center, Department of Radiation Oncology, Somerset, USA ^6^Seattle Cancer Care Alliance Proton Therapy Center, Department of Radiation Oncology, Seattle, USA ^7^Maryland Proton Treatment Center, Department of Radiation Oncology, Baltimore, USA ^8^Mayo Arizona Proton Therapy Center, Department of Radiation Oncology, Phoenix, USA ^9^UF Health Proton Therapy Center, Department of Radiation Oncology, Jacksonville, USA

**Abstract:** Local failure represents a source of morbidity and mortality for patients with locally advanced unresectable or medically inoperable pancreatic cancer (LAPC). We hypothesize that proton therapy (PBT) can achieve durable local control with a reduced risk of side effects as compared to photon therapy.

**Methods:** We analyzed the multicenter prospective registry of the Proton Collaborative Group for patients with LAPC who received definitive PBT. 90% of patients had adenocarcinoma histology, while two patients had either a neuroendocrine tumor or cystadenoma. Overall survival (OS), freedom from local-regional recurrence (FFLR), and freedom from distant metastases (FFDM) was calculated for the adenocarcinoma cohort. Toxicity was calculated for the entire cohort.

**Results:** Nineteen patients were identified. Median age was 70 years. Patients had adenocarcinoma (n=17), neuroendocrine tumor (n=1), or cystadenoma (n=1). Majority had T3-4 (68.4%) disease. Median PBT dose was 54 Gy (IQR: 50.5-59.4). Of patients with adenocarcinoma histology, 76.4% received induction chemotherapy, and 82% received concurrent chemotherapy. Median follow-up time was 10.0 months. Median, and 1-year rates of OS were 13.0 months, 50.8%, respectively. The 1-year FFLR rates was 81.3%. The 1-year FFDM rates were 58.0%. Toxicities were mild and predominantly anorexia (21% Grade 2) or fatigue (21% Grade 2), with no Grade >3 acute or late toxicity.

**Conclusions:** This study shows excellent local control following PBT in LAPC, with a lower side effect profile than in modern IMRT photon series. Additional studies are needed to determine if PBT can further improve outcomes without adding toxicity using dose escalated strategies for LAPC.

## PTCNA-0099

### Simultaneous integrated boost/protection with proton beam therapy for hepatocellular carcinomas

#### Matthew Greer^1^, Stephanie Schaub^1^, Avril O'Ryan-Blair^2^, Tony Wong^2^, Smith Apisarnthanarax^1^

##### ^1^University of Washington, Department of Radiation Oncology, Seattle, USA ^2^Seattle Protons Center, Department of Radiation Oncology, Seattle, USA

**Purpose/Objectives**: We present a retrospective single institution study on the clinical outcomes of patients with hepatocellular carcinomas (HCCs) treated with proton beam therapy (PBT) using a simultaneous integrated boost/protection (SIB/P) technique to dose escalate to tumors while protecting organs-at-risk (OARs).

**Materials/Methods:** Thirty-one consecutive HCC patients were treated with SIB/P PBT between 2014-2020 with a 15-fraction regimen of 45.0-67.5 Gy(RBE). Non-classic radiation-induced liver disease (RILD) was defined by a Child-Pugh (CP) score increase 2+ and/or RTOG grade 3 enzyme elevation. Overall survival (OS), progression-free survival (PFS), and local control (LC) were calculated using the Kaplan-Meier method and univariate predictors of OS by Cox regression analysis.

**Results**: Patients represented a high-risk cohort: 39% with BCLC stage C, 16% with CP-B/C cirrhosis, and a median gross tumor volume (GTV) diameter of 10.2 cm. Pencil beam scanning was used in all patients. An average GTV mean of 62.0 Gy(RBE) was achieved with an average D99 49.5 Gy(RBE) and D95 52.8 Gy(RBE). Median follow-up for all patients was 8 months. 1-year OS and PFS were 53% (95% CI 34-72%) and 75% (95% CI 39-93%), respectively. 1-yr LC was 86.2% (95% CI 63-96%), with two isolated LF. One patient experienced RILD.

**Conclusions:** In this series of HCC patients with high-risk tumors, moderate dose escalated PBT with SIB/P technique that delivers heterogeneous tumor dose results in excellent local control rates and minimal toxicities.

## Clinics: GU PTCNA-0046

### Initial clinical experience of the bladder-filling controls using ultrasound bladder scanner for proton prostate patients

#### Chin-Cheng Chen^1^, Jason Pineiro^1^, Danielle Boos^1^, Shiomo-Kalman Rosenfeld^1^, Andrew Okhuereigbe^1^, Daniel Gorovets^2^, Shaakir Hasan^1^, Haibo Lin^1^

##### ^1^New York Proton Center, Radiation Oncology, New York, USA ^2^Memorial Sloan Kettering Cancer Center, Radiation Oncology, New York, USA

**Purpose:** Consistent daily bladder volumes (BVs) during a course of proton therapy for prostate cancer improves the treatment accuracy and efficiency especially for fixed-beam and SBRT patients. The initial clinical experience of using an ultrasound bladder scanner to optimize bladder-filling was demonstrated.

**Methods:** The CUBEscan^TM^ software used for the BioCon-750 bladder scanner calculates the ultrasound-BV from 12 planes instead of ellipsoid estimation with coronal and sagittal diameters. The daily ultrasound-BV was measured prior to X-ray setup imaging. The patient would wait longer if the ultrasound-BV is less by 25% of the volume calculated in plan CT (pCT) but no void if the ultrasound-BV was larger. The patient-specific drink instruction (16-24 oz., 30-60 mins) could be also adjusted to improve the consistency of bladder filling for the remaining fractions. The daily ultrasound-BVs for 6 patients (5 fixed-beam room, and 1 SBRT) were compared with the volumes calculated in pCT, verification CTs (vCTs), and cone-beam CTs (CBCTs), respectively.

**Results:** Figure 1 displays the daily ultrasound-BVs for a fixed-beam prostate patient with BV of 264.6 ml in pCT and 259.8/157.6/270.3 ml in vCTs. Table 1 listed the daily ultrasound and CBCT BVs for a prostate SBRT patient. Preliminary results showed the average daily ultrasound-BV differences versus pCT-baseline were 20.4%, 18.8%, 28.5%, 22.9%, 15.5% and 16.9%, excluding the BVs lager by 50% of pCT-baseline mostly due to treatment delays.

**Conclusions:** Daily ultrasound-BV <25% different from the pCT-baseline was achievable, which minimizes the number of kV/CBCTs, setup/range uncertainties, and improves treatment efficiency.

## Clinics: Sarcoma PTCNA-0070

### Dosimetric Comparison Between Proton Beam Therapy, IMRT, and 3D Conformal Therapy for Soft Tissue Extremity Sarcoma

#### Brady Laughlin^1^, Michael Golafshar^1^, Matthew Prince^1^, Safia Ahmed^2^, Ivy Petersen^2^, Jonathan Ashman^1^

##### ^1^Mayo Clinic Arizona, Department of Radiation Oncology, Phoenix- AZ, USA ^2^Mayo Clinic Rochester, Department of Radiation Oncology, Rochester- MN, USA

**Purpose:** Proton beam therapy (PBT) may provide a dosimetric advantage in sparing soft tissue and bone while achieving target coverage for extremity soft sarcoma (STS). We compare PBT with intensity modulated radiation therapy (IMRT) and 3D conformal radiation therapy (3D CRT) in treatment of extremity STS.

**Materials/Methods:** Seventeen patients previously treated with PBT were collected for this study. Of these patients, 14 patients were treated with preoperative 50 Gy in 25 fractions and are the subject of this study. IMRT and 3D CRT plans were created to compare against PBT plans. Cumulative DVH data were generated to compare techniques. For the clinical target volume, D2, D95, D98, V50, Dmin, and Dmax were assessed. Dmin, D1, Dmax, Dmean, V1Gy, V5Gy, and V50 Gy were evaluated for the adjacent soft tissue. D1cc, Dmax, Dmean, V35-50 were evaluated for bone.

**Results:** All of the plans achieved satisfactory coverage to the clinical target volume. The PBT plans delivered less dose to uninvolved soft tissue and adjacent bone. The mean dose to the soft tissue was 2 Gy, 11 Gy, and 13 Gy for PBT, IMRT, and 3D, respectively. The mean dose to adjacent bone was 15 Gy, 24 Gy, and 27 Gy for PBT, IMRT, and 3D, respectively.

**Conclusion:** PBT for extremity STS demonstrated superior sparing of uninvolved soft tissue and adjacent bone in comparison to IMRT and 3D. Further analysis will identify for which patients PBT provides the maximum benefit. Assessment of clinical outcomes will determine if the dosimetric advantages of PBT correlate with less toxicity.

## Clinics: Head and Neck PTCNA-0053

### Individual spacers for tongue protection by high-dose particle therapy: first experience

#### Maciej Pelak^1^, Klaus Charvat^2^, Sascha Reindl^2^, Markus Stock^1^, Antonio Carlino^1^, Joanna Gora^1^, Eugen B. Hug^1^

##### ^1^MedAustron, Ion Therapy Center, Wiener Neustadt, Austria ^2^Charvat Medlounge, Dentistry, Theresienfeld, Austria

**Background and Aim**: Acute tongue mucositis and dysgeusia are common toxicities of particle therapy applied in the proximity of oral cavity. In the present report we describe application of individual tongue spacers that may help reduce these side effects by mechanically moving the tongue away from the irradiation field.

**Material and Methods**: Fourteen patients planned for particle therapy for one of the following locations: parotid, nasal cavity, paranasal sinuses or submandibular salivary glands. The spacers were individually made from wax for fitting and finally from cold polymer resin on plaster teeth molds. Shape was determined by planned irradiation field and desired tongue position based on one of four pre-defined reference models (example shown on Figure 1). Subsequently, the patients had their treatment planned and applied with spacers in place. Treatment toxicity was prospectively recorded.

**Results**: For 12/14 patients (85.7%) the treatment was delivered with protons and for 2 (14.3%) with carbon ions. Mean prescription dose was 66 Gy RBE (range: 66 – 76.8 Gy RBE). By the time of results evaluation 11 patients had completed the therapy. In patients for whom planning CT was available with and without the spacers, a reduction of the maximum dose to the tongue up to 22.3 Gy was observed (Figure 2). The spacers were well tolerated. Radiation mucositis of the tongue was not observed in 9/11 patients (81.8%) and 10/11 (90.9%) remained free from dysgeusia.

**Conclusion**: The individual spacers are a promising strategy to reduce tongue-related toxicities by particle irradiation and should be further explored.

## Clinics: Gynecology PTCNA-0043

### Dosimetric comparison of proton versus photon post-hysterectomy pelvic radiotherapy for patients with endometrial cancer treated on an institutional prospective trial

#### Allison Garda^1^, Lindsay Morris^1^, Randi Finley^1^, Sheri Spreiter^1^, Noelle Deiter^1^, Jon Kruse^1^, Shima Ito^1^, Michael Haddock^1^, Ivy Petersen^1^

##### ^1^Mayo Clinic, Department of Radiation Oncology, Rochester, USA

**Purpose:** Adjuvant pelvic radiotherapy improves locoregional control in high risk and advanced stage endometrial cancer. Pelvic radiotherapy is associated with acute urinary, gastrointestinal, and hematologic toxicity, which may be reduced with proton therapy. This study is a dosimetric comparison of intensity modulated proton beam therapy (IMPT) versus volumetric modulated arc therapy (VMAT).

**Methods:** The first 10 patients enrolled on an institutional prospective non-randomized trial of proton or photon post-hysterectomy radiotherapy were included. Patients underwent CT simulation with pelvic immobilization and a rectal balloon. Full bladder, empty bladder, and IV contrast scans were obtained. Comparison plans were generated. Clinical target volumes (CTV) included vaginal cuff, proximal 3 cm of vagina, and pelvic lymph nodes (internal iliac, external iliac, obturator, presacral, and distal common iliac) to the level of L4/L5. Photon planning target volume (PTV) was 5 mm expansion on CTV. Proton optimization target volume was 7 mm on vaginal cuff CTV and 5 mm on nodal CTV. Prescription to the CTV (IMPT) or PTV (VMAT) was 45 Gy (or relative biological effectiveness 1.1) in 25 fractions.

**Results:** FIGO stages were IB (n=2), II (n=1), IIIA (n=2), and IIIC1 (n=5). Three patients were treated with VMAT and remainder were treated with IMPT. Nine patients received adjuvant chemotherapy. Table 1 shows coverage of the PTV (photon) or CTV (proton) and dose to organs at risk. Figure 1 shows comparison plans for one patient.

**Conclusion:** IMPT is associated with reduced low dose to bowel, bladder, and bone marrow. Additional dosimetric comparisons will be conducted as patients enroll.

## PTCNA-0078

### Initial clinical experience of the vaginal dilator used in female pelvis patients for proton therapy

#### Chin-Cheng Chen^1^, Francis Yu^1^, Pingfang Tsai^1^, Julie Moreau^1^, Chavanon Apinorasethkul^1^, Danielle Boos^1^, Andy Shim^1^, Haibo Lin^1^, J. Isabelle Choi^2^

##### ^1^New York Proton Center, Radiation Oncology, New York, USA ^2^Memorial Sloan Kettering Cancer Center, Radiation Oncology, New York, USA

**Purpose:** A vaginal dilator was used in female pelvis proton therapy to reduce the toxicity of vaginal stenosis. Our initial clinical experience is described including simulation, dilator contouring (HU override), plan optimization, bladder filling, daily IGRT, and continuous plan evaluation with verification scan.

**Methods:** Two consecutive patients were treated to the pelvis with proton therapy using a vaginal dilator. The dilator was inserted with a marked stopping point at the entrance. The physical and water-equivalent thickness (relative stopping power of 1.26) were measured and applied. Three fields (LPO/RPO/AP) with multiple-field optimization were used to deliver a simultaneous integrated boost prescription (50.4/42.0 Gy(RBE)). An ultrasound bladder scanner was used to maintain consistent bladder filling prior to X-ray imaging. Daily kV/CBCT was used to align the patient with <5 mm setup tolerance to bony structures. Verification CTs were performed to evaluate the plan robustness.

**Results:** The vaginal dilator was located at the distal dose fall-off between the anal target and bladder. There was slight inter-fraction variation of the dilator angle and up to 7 mm difference of the inserted length. The final verification plans showed an increase of <5 cm^3^ of vagina V_47.88Gy(RBE)_ with the dilator tilted <4°. No significant changes were found in CTV dose coverages.

**Conclusions:** The entrance marker reproduced the length of the vaginal dilator insertion but did not account for rotational positioning. Pre-treatment ultrasound bladder scan improved the consistency of bladder filling and minimized the dose variations. Clinical outcomes and treatment toxicities of the two patients will be followed.

## Multidisciplinary / Other: Flash PTCNA-0088

### Platform for delivery of proton flash radiation research in a mouse model

#### Robert Emery^1^, David Argento^1^, Marissa Kranz^1^, Jon Jacky^1^, Bob Smith^1^, Ning Cao^1^

##### ^1^University of Washington, Radiation Oncology, Seattle, USA

**Background and Aims**: An integrated platform has been created at the University of Washington Medical Cyclotron Facility to conduct proton FLASH research on a mouse model.

**Methods**: A cyclotron beamline has been modified to produce a 6cm diameter scattered beam at dose rates between 0.1 to 100 Gy/s. Dose is monitored using a microDiamond detector connected to a Keithley 6517B electrometer. The diamond detector is calibrated against an Advanced Markus chamber. The electrometer is integrated with the cyclotron control system to deliver the desired dose. A GUI allows researchers to set the dose, deliver beam, and record dose, dose rate, and delivery time without accelerator operator assistance.

A wirelessly controlled, six-axis robotic arm acts as the mouse support and positioning assembly with a 3D printed mouse bed attached as the end effector. The beam is collimated with variable graphite jaw collimators and field shape is verified with a light field.

**Results**: Six irradiation sessions have been conducted irradiating 30 mice per session with both FLASH (60Gy/s) and conventional dose rate (0.5 Gy/s) protons. The time to position a mouse at isocenter and adjust and verify the radiation field shape is on the order of 30 seconds. Conventional rate dose is reproducible to within 0.01 Gy. FLASH dose can vary as much as 2Gy between runs.

**Conclusions**: Mouse positioning and field adjustment is fast and user-friendly. Work is currently underway to improve light field and collimator accuracy. A new electrometer from Pyramid Technical Consultants is being investigated to improve FLASH dose reproducibility.

## Multidisciplinary / Other: Facility administration, operations and set up PTCNA-0055

### Survey of Session Times by Site for Two Single Room Proton Centers

#### Rex Cardan^1^, Grant Evans^2^, Charles Shang^2^, James W Snider^1^, Richard Popple^1^

##### ^1^University of Alabama at Birmingham, Radiation Oncology, Birmingham, USA ^2^SFPTI Proton Center, Radiation Oncology, Delray, USA

**Purpose**: The high cost of proton treatment centers can result in unsustainable economic pressure if the facility is not planned appropriately. Single room centers provide a lower cost facility with a tradeoff of a planning to treat at the maximum capacity. It is critical then to understand realistic treatment times for the patient population served. We analyzed treatment times from two single room proton centers to obtain site specific expectations.

**Materials and Methods**: Database queries were performed from two independent facilities A) a private center treating predominately prostate, and B) a large academic medical center treating a complex case distribution. Treatment sites were grouped by 2 field prostate (2FP), 3 field prostate (3FP), brain (BRN), 3 field head/neck (3FHN), 4+ field head/neck (4F+HN), spine (SPN) and breast/chest wall (BCW). Sites were identified retrospectively using plan names. Session time was defined as the time of the first image to the last beam off time.

**Results**: 541 patient datasets encompassing 5737 sessions were analyzed. The average session times were 8.0 ± 2.2 min, 11.6 ± 5.6 min, 13.7 ± 6.8 min, 12.3 ± 4.8 min, 12.3 ± 5.4 min, 16.7 ± 10.9 min, 16.8 ± 5.2 min for 2FP, 3FP, BRN, 3FHN, BCW, SPN, and 4F+HN respectively. The overall session times were 11.2 ± 8.5 min and 19.7 ± 9.3 min for Facility A and B respectively.

**Conclusion**: The analyzed data in this study provide a reasonable collection of treatment data to plan future centers for various patient populations.

## PTCNA-0095

### Evidence-Based Practice Improvement in Radiation Oncology

#### K. Halda^1^, L. Fong de los Santos^1^

##### ^1^Mayo Clinic, Radiation Oncology, Rochester, USA

**Background:** Implementing a web-based system to capture events, incidents, and areas that need improvement has led to a significant number of project improvements and benefited the culture of safety.

**Method and Materials:** Mayo Clinic has executed a **Sa**fety, **I**mprovement, and **L**earning **S**ystem (SAILS). This has become a platform that is reviewed routinely to learn about what we can do to improve safety and provide high-quality service. Construction of the form, input from employees, and steps for reviewing cases has been ongoing and successful in the project improvement initiatives.

**Results:** Since implementing SAILS we have seen an increase in documentation and action from miss and near miss situations in radiation treatments and plans, in protons and photons. This has also been improved non-modality-related occurrences in treatment scheduling, consults, etc.

**Conclusions:** Having an electronic, multiple-step program and process has led to timely identification of process gaps, recommendations, and improvements in all areas of our Radiation Oncology practice.

## Physics: Quality Assurance & Verification PTCNA-0084

### Feasibility study of utilizing XRV-124 scintillation detector for collinearity measurement in uniform scanning proton therapy

#### Colton Eckert^1^, Biniam Tesfamicael^1^, Michael Chacko^1^, Hardev Grewal^1^, Suresh Rana^1^

##### ^1^Oklahoma Proton Center, Medical Physics, Oklahoma City, USA

**Purpose**: The purpose of this study was to determine the feasibility of utilizing XRV-124 scintillation detector in measuring the collinearity of the X-ray system and uniform scanning proton beam.

**Methods**: A brass aperture for Snout 10 was manufactured. The center of the aperture had an opening of 1 cm in diameter. The 2D kV X-ray images of the XRV-124 were acquired such that the marker inside the detector is aligned at the imaging isocenter. After obtaining the optimal camera settings, a uniform scanning proton beam was delivered for various ranges (12 g/cm^2^ to 28 g/cm^2^ in step size of 2 g/cm^2^). For each range, 10 monitor units (MU) of the first layer were delivered to the XRV-124 detector. Collinearity tests were then repeated by utilizing EDR2 and EBT3 films following our current QA protocol in practice. The results from the XRV-124 measurements were compared against the collinearity results from EDR2 and EBT3 films.

**Results**: The collinearity results were evaluated in the horizontal (X) and vertical (Y) directions. The average collinearity in the X-direction was -0.24±0.30 mm, 0.57±0.39 mm, and -0.27±0.14 mm for EDR2, EBT3, and XRV-124, respectively. The average collinearity in the Y-direction was 0.39±0.07 mm, 0.29±0.14 mm, and 0.39±0.03 mm for EDR2, EBT3, and XRV-124, respectively.

**Conclusion**: On average, the results from the XRV-124 had a better agreement with that of EDR2. The use of XRV-124 for collinearity tests in uniform scanning protons can improve the efficiency of the QA workflow compared to the films.

## PTCNA-0089

### Analysis of patient QA results comparing RayStation TPS predicted vs. measurements for uniform scanning proton therapy

#### Biniam Tesfamicael^1^, Colton Eckert^1^, Hardev Grewal^1^, Michael Chacko^1^, Suresh Rana^1^

##### ^1^Oklahoma Proton Center, Medical Physics, Oklahoma City, USA

**Purpose**: The objective of the current study was to present the comprehensive patient-specific quality assurance (QA) results by comparing RayStation treatment planning system (TPS) predicted dose vs. measured dose in uniform scanning proton therapy.

**Methods**: Proton plans of various disease sites were generated in RayStation TPS. The disease sites studied include abdomen, bladder, bowel, brain, breast, chest wall, esophagus, larynx, liver, mediastinum, head and neck, pelvis, prostate, sacrum, and spine. The field size ranged from 3 cm to 28 cm, whereas the proton beam range and modulation ranged from 4 to 31 g/cm^2^ and 2 to 19 cm, respectively. Measurements were acquired using a parallel plate ionization chamber in a water tank following the institution's QA protocol. The TPS predicted results calculated based on an in-house developed output factor model were then compared against the measurements.

**Results**: A total of 705 proton fields were irradiated. The differences between predicted vs. measured doses were the following – abdomen: 0.10±0.36%; bladder: -0.47±1.15%; bowel: 0.81±1.04%; brain: 0.28±1.03%; breast: 0.88±1.24%; chest wall: 2.09±1.31%; esophagus: 0.39±1.26%; larynx: 0.47±0.68%; liver: 0.01±1.02%; mediastinum: 0.28±0.56%; head and neck: 0.23±0.74%; pelvis: -0.40±0.73%; prostate: -0.80±0.60%; sacrum:0.17±0.55%; spine: 0.31±0.48%.

**Conclusion**: Overall, 93.9% of proton fields were within ±2% and did not require monitor units (MU) adjustment. For measurements outside of ±2%, 6.1% of proton fields were recalibrated with the measured MU. The major discrepancies between predicted and measured dose were seen for the breast and chest wall patients.

## PTCNA-0104

### Fast proton beam fluence and position detector array with multi-coordinate readout

#### Evgeny Galyaev^1^, Pablo Camacho^2^, Aleksei Krasilnikov^2^, Martin Bues^3^, Jiajian Jason Shen^3^, Rafael Acuna Briceno^4^

##### ^1^Radiation Detection and Imaging RDI Technologies, Physics, Tempe, USA ^2^Radiation Detection and Imaging RDI Technologies, Electronics, Tempe, USA ^3^Mayo Clinic College of Medicine and Science, Radiation Oncology, Phoenix, USA ^4^Arizona State University, Ira A. Fulton Schools of Engineering, Tempe, USA

Using Pencil Beam Scanning (PBS), treatment plans are fully described by a set of beam spot parameters such as energy, time duration, position, angle, etc. Even as PBS has become the new standard in proton radiotherapy, most quality assurance instrumentation has not been designed to complement PBS. A detector capable of measuring beam parameters spot by spot in real time would enable richer diagnostics and further restriction on proximal margins. A new planar detector array capable of recording proton beam position and fluence at a rate of 25kHz has been built, and is now being characterized. With sub-millimeter resolution in beam positioning, the new device overcomes most common limiting performance factors of planar detector arrays with a conventional pixelated arrangement of sensors. A large-area (1,260cm2) array proof-of-concept is also nearing its completion. Gas ionization is collected by a planar arrangement of strips projected along three directions in a beam transverse plane, from which beam shape (covariance) and size, as well as position, are reconstructed for each recorded data frame. Such proposed multi-directional readout provides a large, isotropic and continuous active area, while using fewer data channels (vs. pixel-based arrays). Combined with a novel approach to tomographic reconstruction, the updated preliminary experimental results demonstrate spatial resolution of better than 200μm and down to 100μs timing resolution. These findings open additional avenues to the enhanced machine and patient level quality assurance of the PBS as well as continuous line-scanning proton beam modalities through its superior timing capabilities, coordinate resolution and dose precision registration.

## Physics: Treatment planning PTCNA-0052

### A clinical comparison of two commercial treatment planning systems for spot-scanning proton therapy

#### Daniel Mundy^1^, Thomas Bradley^1^, Ashley Hunzeker^1^, Luis Fong de los Santos^1^, Jon Kruse^1^, Janelle Miller^1^, Alan Kraling^1^, John Antolak^1^, Eric Brost^1^, Anita Mahajan^1^

##### ^1^Mayo Clinic, Radiation Oncology, Rochester, USA

**Purpose:** Treatment planning for proton therapy is evolving rapidly with the additions of multi-criterion optimization, GPU-based calculation, LET-based optimization, etc. to commercial treatment planning systems (TPS). As the needs of a clinical practice also evolve, it becomes necessary to periodically re-evaluate available options. In this study, we evaluated RaySearch's RayStation against our existing Varian Eclipse TPS.

**Methods:** A core group of physicists, dosimetrists, and physicians compiled a list of functionalities to evaluate using an on-site test system. Testers were provided training and support from RaySearch such that unfamiliarity with the new software would not hinder evaluation. Functionalities were ranked by importance for patient care (IPC) and scored based on a performance index (PI) according to evaluation metrics previously developed in our clinic for evaluation of oncology information systems.

**Results:** In Table 1, Advantage indicates whether the PI favored Eclipse (blue) or RayStation (red) and the Test Score represents performance weighted by IPC, where a perfect score would be 100%. Of the 24 features tested, 7 favored Eclipse, 11 favored RayStation, and 6 were neutral. With Test Scores below 60%, neither TPS is ideal. Based on PI, 16 features were identified as being acceptable and 8 unacceptable for both systems (Fig. 1).

**Conclusion:** The Eclipse and RayStation systems were found to be comparable to one another, each with advantages and disadvantages and neither being an ideal solution. This study, which spanned more than a year from initiation to completion, also highlighted the complexities of software evaluation with the increasing complexity of IT environments.

## PTCNA-0098

### Comparison of 3D conformal neutron therapy with proton boost versus intensity modulated neutron therapy for salivary tumors

#### Matthew Greer^1^, Natalie Viscariello^1^, Upendra Parvathaneni^1^, Jay Liao^1^, George Laramore^1^, Robert Stewart^1^

##### ^1^University of Washington, Department of Radiation Oncology, Seattle, USA

**Purpose**: Treatment of locally advanced salivary gland tumors with skull base invasion is a major clinical challenge, due to the dose limits of adjacent critical structures. One effective treatment approach is to combine a 3D conformal fast neutron therapy (3DCNT) treatment with a proton boost to the skull base. Recent technical advances have enabled intensity-modulated neutron therapy (IMNT) at the University of Washington. We evaluated the dosimetry of IMNT as an alternative to the combined 3DCNT with a proton boost with locally advanced salivary gland tumors with skull base invasion.

**Methods**: Two patients treated with 3DCNT and a proton boost for adenoid cystic carcinoma of palate with skull base invasion were retrospectively replanned using IMNT. Patients received 18.4Gy in 16fx 3DCNT (equivalent to 74 Gy in 37fx of x-rays) with a subsequent 30 Gy proton boost delivered in 15fx to the skull base. Dose volume histograms (DVHs) were used for plan comparison.

**Results**: Table 1 below compares relevant changes in dosimetry for the IMNT and 3DCNT+proton plans. Planning target volume (PTV) coverage was equivalent for both treatment approaches. The IMNT plan allowed for significant sparing of adjacent optic structures and the temporal lobe.

**Conclusions**: IMNT achieved comparable target volume coverage compared with high-LET neutrons, while significantly reducing dose to adjacent tissues compared to the 3DCNT + proton boost. IMNT has the potential to reduce treatment toxicity and improve other quality of life metrics (e.g., treatment duration) compared to 3DCNT with a proton boost.

